# Second-Pass Vessel Arrangement and Separation-Limited Operation in Two-Pass Seawater Reverse Osmosis Under Progressive Fouling: Implications for Electrolytic Hydrogen Production

**DOI:** 10.3390/membranes16090290

**Published:** 2026-08-28

**Authors:** Tomas Rojas, Pablo Cassorla, Adrian Rojas, Gonzalo Aguila

**Affiliations:** 1 Department of Chemical and Environmental Engineering, Universidad Técnica Federico Santa María, Santiago 8940897, Chile; 2 Remote Waters SpA, Santiago 7860340, Chile; 3 Technology Center for Packaging Innovation (LABEN-CHILE), Department of Science and Food Technology, Faculty of Technology, University of Santiago of Chile (USACH), Santiago 9170201, Chile; 4 Independent Researcher, Santiago 7810803, Chile

**Keywords:** seawater reverse osmosis, membrane fouling, pressure vessel arrangement, specific energy consumption, process modelling, two-pass configuration, water electrolysis, energy recovery device

## Abstract

Seawater reverse osmosis (SWRO) supplies the purification chain of electrolytic hydrogen plants, yet its long-term behaviour under fouling is rarely resolved at train level. This work presents a dynamic, quasi-steady-state model of a two-pass SWRO train and compares three second-pass architectures under progressive first-pass fouling, represented by time-evolving water- and solute-permeability coefficients. At each of 51 instants over a 500-day horizon, the operating point is re-optimized to minimize specific energy consumption (SEC), subject to a product limit of 20 mg·L^−1^ set by the downstream polishing stage rather than the electrolyzer. Two share-membrane types, element count and installed area, differ only in vessel arrangement; the third is the industrial reference. Arrangement alone changes SEC by 36.9–41.6% across three fouling scenarios, 8.38 against 11.46 kWh·m^−3^ under severe fouling, widening to 71% at fixed production. Fouling constrains the system through separation, not hydraulics: the technically admissible operating window widens as membranes degrade, whereas the window satisfying the product specification closes. The energy penalty of compliance begins at days 310 and 170, respectively, accumulating about twice as fast thereafter in the former. Energy recovery reduces SEC by 63–64% without altering the ordering.

## 1. Introduction

The pressing global need to counteract human-induced climate change has spurred a profound transformation in the energy sector, elevating green hydrogen to a pivotal role as an energy carrier in the pursuit of carbon neutrality. Green hydrogen, produced through water electrolysis powered by variable renewable sources such as wind and solar, offers a flexible strategy for decarbonizing challenging sectors, including heavy industry, chemical manufacturing, and long-distance shipping [1]. Yet, as countries set ambitious electrolysis capacity targets, the sustainability of this evolution is increasingly assessed in terms of the water–energy interplay. While early techno-economic analyses prioritized minimizing renewable electricity costs, the supply and purity of water feedstock have become critical determinants of operational reliability and economic viability for hydrogen production facilities [2]. Far from being solely energy-intensive, green hydrogen production is equally water-demanding, requiring significant quantities of ultrapure water to ensure the durability of electrochemical components [2]. Water electrolysis requires feed water that meets at least ASTM D1193 Type II specifications (corresponding to a resistivity of ≥1 MΩ·cm and a conductivity below 1.0 μS/cm), a standard that effectively precludes the direct use of any untreated natural water source [3,4].

That requirement is imposed not on a single treatment unit but on a chain of them, and the distinction governs the design of the desalination stage. Modern water electrolysis technologies, specifically Proton Exchange Membrane (PEM) and Alkaline Water Electrolyzers (AWE), are highly sensitive to contaminants in the feed water. To prevent performance degradation, the feed water must adhere to stringent standards, such as ISO 3696 [5] Grade 2 or ASTM D1193 Type II, which specify a conductivity of less than 1.0 μS/cm [2], and commercial manufacturers commonly specify a feed conductivity below 1 μS/cm for their electrolyzers [6]. In laboratory and pilot-scale operations, achieving this level of purity is often handled by specialized systems, but for industrial-scale production, integrated water treatment plants are required. No single desalination stage delivers water of that quality from seawater. The purification train adopted in practice is therefore sequential: reverse osmosis (RO) removes the bulk of the dissolved salts, and a final polishing stage—ion exchange or continuous electrodeionization—performs the last demineralization to electrolysis grade [3,7]. Deionization and purification can account for a significant portion of the balance-of-plant costs, often representing up to 22% of the total capital expenditure for 1 MW systems [2]. Furthermore, the spatial and temporal profiles of potential within the electrolyzer are influenced by the local ionic environment, and membrane fouling and progressive degradation of permeability coefficients along the pressure vessel directly compromise compliance with these conductivity thresholds, meaning that even minor deviations in water resistivity over time can lead to uneven current distribution and accelerated material wear [8].

Each link of that chain carries its own inlet specification, and it is the polishing stage, not the electrolyzer, that sets the target the desalination train must meet. Continuous electrodeionization requires a feed below 25 mg·dm^−3^ of total dissolved solids (TDS) [9], equivalently a feed conductivity below 40 μS/cm, or of the order of 20 mg·L^−1^ of dissolved solids [10]. This requirement follows from the ion-exchange resin and from the electrical regeneration of the module, and is therefore independent of the origin of the feed water. The present work adopts 20 mg·L^−1^ as the product specification of the seawater reverse osmosis train, which is conservative with respect to the reported polishing-inlet values. The question addressed here is accordingly not whether a reverse osmosis train can reach electrolysis-grade water on its own, which it cannot, but for how long and at what energy cost it can keep delivering water within the specification of the stage that follows it, as its membranes progressively foul.

The mechanisms of degradation caused by water impurities are complex and multifaceted. In PEM electrolyzers, the polymer electrolyte and the catalyst-coated membranes are particularly vulnerable to poisoning by foreign cations [2]. Cationic species such as Fe^3+^, Cu^2+^, and Al^3+^, commonly found in insufficiently treated water, migrate into the membrane and occupy sulfonic acid sites, thereby displacing protons and reducing the ionic conductivity of the electrolyte [11]. This displacement increases the cell’s ohmic resistance, leading to a dramatic initial drop in performance, followed by a slower, cumulative degradation [11]. Beyond ohmic losses, certain impurities (such as iron) can react with hydrogen peroxide, a byproduct of the oxygen evolution reaction, to form aggressive hydroxyl and hydroperoxyl radicals [12]. These radicals facilitate chemical thinning of the membrane through fluoride emission and structural bond cleavage, ultimately leading to gas crossover and potential safety failures [12]. Recent studies have investigated the impact of tap water cations at various concentrations, revealing that performance losses can be partially recovered through current-driven processes, though long-term durability remains compromised [13].

In regions with high renewable energy potential and severe freshwater scarcity, Seawater Reverse Osmosis (SWRO) has emerged as the primary technological solution for producing high-purity water feedstock for electrolysis [14]. SWRO is favoured for its modularity and relatively low specific energy consumption (SEC) compared to thermal desalination methods. To achieve sub-ppm total dissolved solids levels required for electrolysis, SWRO systems commonly employ multi-pass or multi-stage configurations [14]. These advanced designs are essential for processing diverse feed sources, including high-salinity seawater, to achieve a consistent permeate quality of 0.028–0.5 ppm TDS [14], although these values correspond to particular configurations, feed conditions, and operating points rather than to the general performance of a two-pass train, and they are not assumed here: the system modelled in the present work is required only to satisfy the polishing-inlet specification defined above. The integration of these systems is particularly relevant in coastal areas; for example, techno-economic analyses in Scandinavian contexts suggest that stand-alone multi-stage RO systems can produce ultra-pure water at costs as low as $0.69 m^−3^, significantly lower than hybrid systems incorporating electrodeionization [15]. However, the two are not substitutes in the train considered here, since the polishing stage performs a demineralization that the reverse-osmosis stage cannot.

However, the energy footprint of desalination remains a concern, as purifying seawater requires 2.5–4.0 kWh of electricity per cubic metre of freshwater produced under typical plant operating conditions [16]. In the context of green hydrogen, this energy represents an additional overhead that must be minimized to keep the levelized cost of hydrogen competitive. While the specific energy consumption for water supply is relatively low compared to the energy required for electrolysis itself (often less than 0.15% of the total energy input), the operational reliability of the desalination plant is paramount [17]. For offshore platforms, water management is often underestimated and requires sophisticated treatment loops to address the unique challenges of the marine environment [18]. Cooperation between offshore wind and RO systems offers a promising pathway to address both freshwater scarcity and hydrogen production by leveraging surplus renewable power to drive high-pressure pumps and energy-recovery devices [19]. A feature of reverse osmosis that bears directly on how such systems are compared is their modular character: plant capacity is reached by replicating trains rather than by scaling a single one, and implemented designs with variable output consist principally of several trains operated in parallel [20,21,22]. The performance of a single train is therefore the unit in which architectures should be compared, and plant-level requirements follow from it by replication.

The most significant barrier to the sustainable and efficient operation of RO systems is membrane fouling, often described as the technology’s “Achilles heel” [23]. Fouling involves the accumulation of undesirable materials on the membrane surface or within the feed channel spacers, leading to various operational problems [20]. These include reduced production capacity, increased energy consumption, and decreased produced water quality [23]. Fouling is broadly categorized into four main types: particulate/colloidal fouling, inorganic scaling, organic fouling, and biofouling [24]. Biofouling, caused by bacterial growth and the formation of resilient biofilms, is considered the most difficult to manage because microorganisms can thrive even in low-nutrient environments [25]. The result of this accumulation is an increase in the required feed pressure to maintain a constant flux, leading to higher operational costs and more frequent membrane replacements [26].

The presence of a fouling layer also exacerbates concentration polarization, a phenomenon in which the concentration of solutes at the membrane surface is significantly higher than in the bulk solution [27]. This increases normalized salt passage, compromising permeate purity and potentially allowing harmful ionic contaminants to enter the electrolyzer feed stream [28]. From an operational perspective, the coupling between fouling progression and permeate quality is particularly critical in SWRO systems designed for green hydrogen: as the water permeability coefficient (A) declines and the solute permeability coefficient (B) increases with fouling time, both the specific energy consumption and the ionic conductivity of the product water evolve in ways that conventional static models fail to capture [8]. Research has shown that as the fouling factor decreases from 1.0 to 0.6, specific energy consumption can increase by 41–50%, depending on the membrane material, while permeate salinity can increase by more than 126% [29]. Crucially, the magnitude of these penalties is not fixed by the fouling alone but is mediated by the system architecture, since high recovery rates—while desirable for maximizing water yield—also increase the potential for inorganic scaling, particularly in the final elements of the pressure vessel, where feed salinity is highest, and ion saturation indices for CaCO_3_ and CaSO_4_ are most unfavourable [30]. This sensitivity of the fouling penalty to the chosen configuration is central to the present study.

Effective mitigation of fouling requires robust pretreatment strategies and advanced modelling tools. Conventional pretreatment methods, such as coagulation and media filtration, are increasingly being replaced or supplemented by membrane-based technologies, such as ultrafiltration (UF) and microfiltration (MF) [31]. UF is particularly effective at removing suspended solids and macromolecules, thereby extending the lifespan of downstream RO membranes [32]. However, the choice of disinfection technique also plays a role; chlorination, while common, can damage polyamide RO membranes and release assimilable organic carbon that fuels subsequent biofouling [33,34]. To mitigate scaling and biological fouling simultaneously, green inhibitors used as antiscalants and antibacterial agents are being explored as an environmentally friendly alternative to traditional chemical treatments [35].

Beyond pretreatment, advanced mathematical models are increasingly used to predict the long-term performance decline of RO plants, dividing degradation into initial compaction stages and more stable fouling stages [36]. The functional form of that decline has been examined directly: a review of predictive approaches compiles the laws proposed for the evolution of the water permeability coefficient [37], which range from linear decay to power-law forms and to superpositions of exponentials separating an initial rapid stage from a slower subsequent one [36]. Long-term field studies provide the magnitudes against which such laws are calibrated; a full-scale seawater plant operated over four years reported a decline of about 30% in water permeability together with an increase of about 70% in solute permeability [38]. Hybrid modelling approaches and artificial intelligence (specifically deep reinforcement learning) are also being applied to optimize RO operation in real time by adjusting parameters to minimize fouling-induced energy losses [39,40]. Recent modelling work has examined how the position of individual spiral-wound modules within a pressure vessel modulates the impact of permeability loss on the specific energy consumption at the minimum-SEC operating point [8]. Alongside the temporal question, a body of work has established the analysis of operating windows: the identification of the operating points that satisfy the manufacturer’s constraints, the selection among them of the point of minimum specific energy consumption, and the comparison of alternative vessel configurations on that basis [8,41]. More recently, superstructure-based multi-objective optimization has compared multiple RO configurations on energy, cost, and environmental criteria [42], although under steady-state design conditions rather than progressive fouling. What these approaches have in common is that the operating window, and the configuration ranking that follows from it, are evaluated for a membrane in a fixed state. How that window evolves as the membranes degrade, and, in particular, whether the constraint that eventually binds is hydraulic or one of separation, has not, to our knowledge, been resolved. The practical minimum energy use of these facilities is continually refined through the selection of high-performance membranes, such as the high-rejection SW30HR or high-flux XLE models, and by optimizing system configurations [43,44].

As the green hydrogen industry matures, a systems-level approach that integrates water treatment and electrolysis performance is essential for achieving long-term sustainability and operational resilience [45,46]. This work addresses how the architecture of the second pass of a two-pass SWRO train governs its response to progressive membrane fouling when the train supplies the polishing stage of an electrolytic hydrogen plant. Because electrolyzer performance and longevity are highly sensitive to water purity—with even minor ionic contaminants significantly increasing overpotentials and catalyst degradation rates [2,47]—both the design and the temporal operation of the treatment system are decisive.

The comparison is constructed to isolate the effect of the vessel arrangement. Two of the three second-pass architectures examined employ the same membrane element, the same number of elements, and the same installed membrane area, and differ only in how the vessels are connected: one places two vessels in parallel discharging to a third in series, the other places three vessels in pure parallel. Any difference in performance between them is therefore attributable to the arrangement and to nothing else. The third architecture, built on a larger head element and the conventional choice for the second pass of two-pass trains, is retained as an industrial reference and is not part of the controlled comparison, since it differs also in element size and installed area. The three are industrially representative arrangements rather than a survey of the design space, and the extension to a larger number of vessels and to other topologies is identified as future work.

On that basis, the study resolves three questions that require the temporal dimension to be posed at all. The first is how much of the energy performance of the train is attributable to the vessel arrangement alone, once membrane type and installed area are held fixed. The second is which constraint eventually binds as the membranes degrade: the analysis distinguishes the operating window admitted by the manufacturer’s technical limits from the narrower window that also satisfies the product specification, and follows both over time. The third is the energy cost of remaining within specification, quantified as the difference between the minimum specific energy consumption with and without the product constraint imposed, an indicator that is non-negative by construction and that reports both when compliance begins to cost and how fast that cost accumulates thereafter. The analysis is carried out under three fouling severities and three functional forms of the degradation law, with an energy-recovery device integrated into the selection of the operating point rather than applied afterwards, and with the numerical uncertainty of the model quantified separately from the uncertainty of the fouling parameters.

## 2. Materials and Methods

This section describes the quasi-steady-state model developed to assess how progressive membrane fouling affects the operational viability of a two-pass seawater reverse osmosis (SWRO) train supplying the water-treatment chain of a proton-exchange-membrane (PEM) electrolysis plant. The model couples an element-by-element solution–diffusion description of each pressure vessel with a time-dependent fouling law and a constrained operating-point selection procedure. Three alternative Pass-2 configurations (C1, C2, and C3) are evaluated under identical first-pass and feed conditions. All simulations were implemented in MATLAB (R2024b; The MathWorks, Natick, MA, USA), using the Optimization Toolbox for the nonlinear solver. Membrane element properties were obtained from the manufacturer’s projection software Water Application Value Engine (WAVE v1.83; DuPont Water Solutions, Wilmington, DE, USA); the modelled elements are the commercial FilmTec™ products SW30HR-380, XLE-440 and XLE PRO-4040 of the same manufacturer.

This is a computational study: no experiments were performed, and there are accordingly no reagents to report. However, the study does have materials, in the sense that the modelled train is built from commercial spiral-wound membrane elements whose properties determine every result reported here. Their transport coefficients, active area, element count per vessel and manufacturer operating limits are listed in Table 1, Table 2 and Table 3 of Section 2.5, and each value is traceable either to the corresponding product data sheet or to the calibration against the manufacturer projection software described in Section 2.2.

The system boundary of the model ends at the outlet of the second pass. The polishing stage that follows it, whether ion exchange or continuous electrodeionization, is not modelled, and the product of the train is specified against the feed requirement of that stage rather than against the electrolyzer itself, as set out in the Introduction. Seawater intake, pretreatment, external interconnecting piping and post-treatment likewise lie outside the boundary. These are excluded because they are common to the three architectures compared and would displace all reported energy figures by a similar amount without affecting the comparison between them; the exclusion is revisited among the limitations in Section 4.9.

### 2.1. System Configurations and Design Rationale

The first pass is a conventional SWRO stage and is identical in the three configurations; it comprises two SW30HR-380 pressure vessels operating in parallel, each loaded with four membrane elements, and is represented by a single vessel whose extensive outputs (permeate and reject flow rates, pump power) are scaled by a factor of two. Because both vessels receive the same feed at the same pressure, their solutions are identical, and the scaling is exact rather than an approximation. The design question addressed in this work therefore concerns the second pass, which reduces the salinity of the combined Pass-1 permeate to the level required at the inlet of the downstream polishing stage. Neither pass includes an energy-recovery device in the base case; the effect of adding one is examined in Section 4.3. Three arrangements of the second pass are compared:

- Configuration C1. Pass 2 is arranged as two vessels in series: vessel V2A (XLE-440, four elements) receives the full Pass-1 permeate, and vessel V2B (XLE PRO-4040, two elements) is fed by the V2A concentrate at its residual pressure. The final product is the sum of the V2A and V2B permeate streams. Installed membrane area: 179.76 m^2^.
- Configuration C2. Vessels V2A and V2B (XLE PRO-4040, two elements each) operate in parallel, each fed with half of the Pass-1 permeate; their combined concentrate then feeds a third vessel, V2C (XLE PRO-4040, two elements), in series. The final product is the sum of the V2A, V2B, and V2C permeate streams. A feed-flow compatibility check ensures that the combined concentrate to V2C does not exceed the element’s maximum feed flow. Installed membrane area: 48.48 m^2^.
- Configuration C3. Vessels V2A, V2B, and V2C (XLE PRO-4040, two elements each) operate in pure parallel, each fed with one-third of the Pass-1 permeate, and their three concentrates are discarded. The final product is the sum of the three permeate streams. Installed membrane area: 48.48 m^2^.

**Figure 1:**
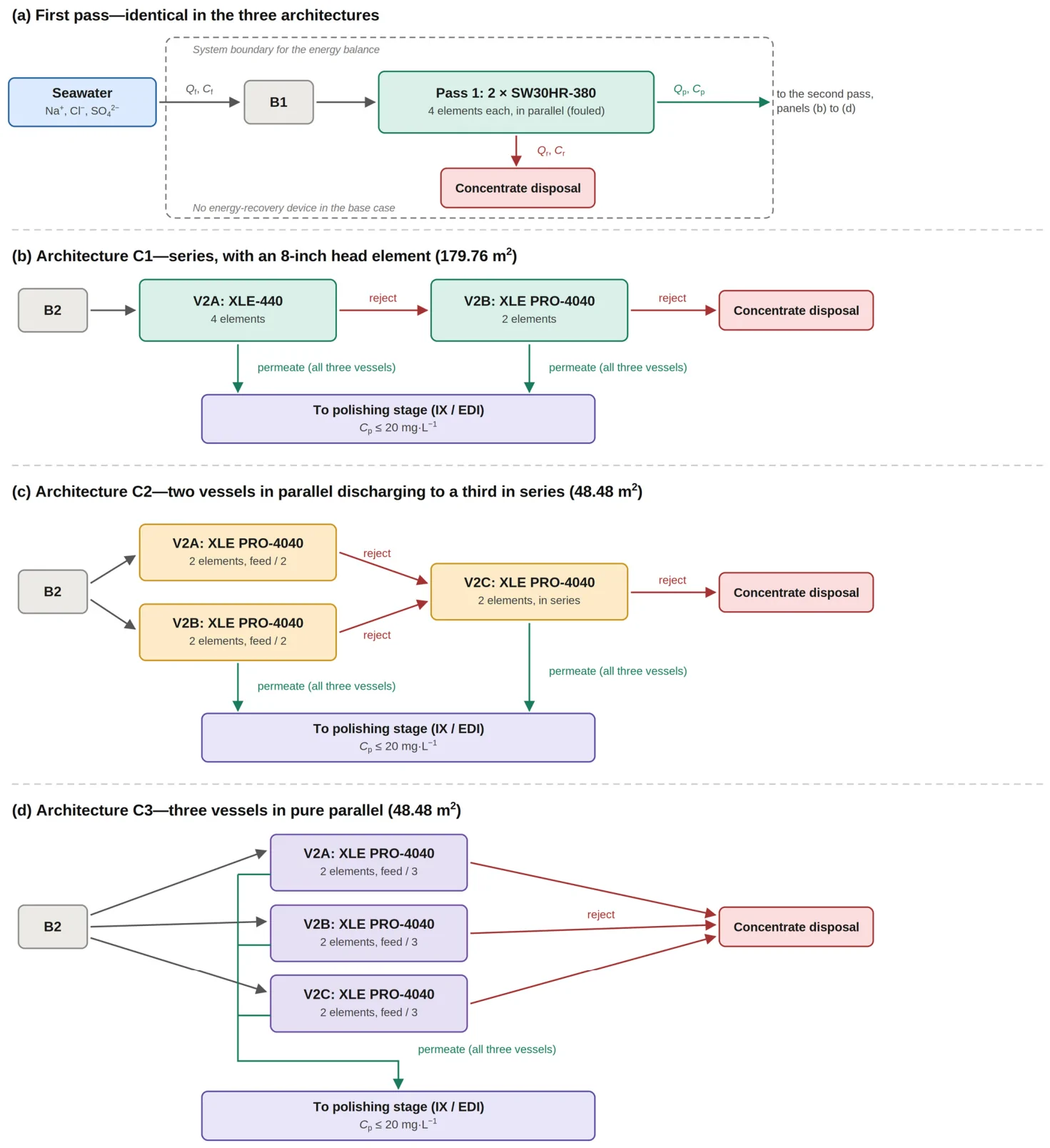
Schematic of the two-pass seawater reverse osmosis (SWRO) train: (**a**) Pass 1, shared by the three configurations (pump B1 feeding two SW30HR-380 vessels in parallel; permeate to Pass 2, reject to disposal); (**b**) configuration C1, with Pass-2 vessels V2A and V2B in series, the V2A reject feeding V2B and only the V2B reject sent to disposal; (**c**) configuration C2, with V2A and V2B in parallel (each receiving half of the Pass-1 permeate) and their combined reject feeding V2C in series; (**d**) configuration C3, with V2A, V2B and V2C in pure parallel, each receiving one third of the Pass-1 permeate and all three rejects sent to disposal. In every configuration, the permeate streams from all Pass-2 vessels combine to form the product water delivered to the downstream polishing stage, and only the final-vessel reject is discharged. The system boundary of the energy accounting is indicated in panel (**a**); no energy-recovery device is present in the base case.Throughout, dark-grey arrows denote feed streams, green arrows denote permeate streams and red arrows denote reject (concentrate) streams; the grey dashed line marks the system boundary of the energy accounting. Box colours are used only to distinguish elements visually and carry no additional meaning.

The comparison between C2 and C3 is controlled: the two employ the same membrane element, the same number of elements, and the same installed area, so that any difference between them is attributable to the arrangement of the vessels alone. C1 differs additionally in element size and installed area, and is retained as the industrial reference, being the arrangement that constitutes standard practice for the second pass of two-pass SWRO trains. It is not part of the controlled comparison and is reported as such throughout. The aim of the comparison is not to advocate one arrangement over the other, but to characterize how each responds to progressive fouling and to expose any difference in performance between them. The three arrangements are industrially representative rather than a survey of the design space; the extension to a larger number of vessels and to other topologies is future work. The shared first pass and the three second-pass configurations are shown schematically in Figure 1.

The two passes use membranes matched to their feed salinity. Pass 1 employs seawater (SW) membranes (SW30HR-380), suited to the high osmotic pressure of raw seawater, whereas Pass 2 polishes an already partially desalinated, low-salinity stream and therefore uses low-energy brackish-water-type membranes of the XLE family (XLE-440 and XLE PRO-4040). These operate at substantially lower feed pressure and energy demand, which is standard practice for the second pass of two-pass SWRO trains and is appropriate given the reduced salinity of the Pass-1 permeate.

Each pass is driven by a single high-pressure pump (B1 for Pass 1, B2 for Pass 2); downstream vessels recover the residual pressure of the upstream concentrate without an additional pump. The principal modelling assumptions are as follows:

- Fouling is applied to Pass 1 only; Pass-2 membranes are assumed unfouled over the simulated horizon. The justification is developed in Section 2.3, and the robustness of the results to this boundary is tested by imposing degradation on the second pass as well.
- No reject recirculation is considered.
- Each vessel is solved element by element, propagating the feed composition and flow rate from one element to the next.

#### Connectivity Relations Between Vessels

The element-level equations of Section 2.2 describe transport within a vessel, whereas what distinguishes the architectures is how the vessels are connected. In the three configurations, the stream entering the second pass has flow rate Q0 = 2Qp,P1 and composition C0,i = Cp,P1,i, where P1 denotes the permeate of a single first-pass vessel, and the feed pressure of every lead second-pass vessel is the discharge pressure of pump B2. A vessel placed in series receives, as feed, the concentrate of the vessels upstream of it, at the pressure those vessels deliver, that is, their feed pressure less their accumulated pressure drop.

In C1, vessel V2A receives (Q0, C0,i) and V2B receives the V2A concentrate, (Qr,V2A, Cr,V2A,i), at pressure PB2 − ΔPV2A, so that the product is Qprod = Qp,V2A + Qp,V2B with the composition of the flow-weighted mixture of the two permeates. In C2, vessels V2A and V2B each receive (Q0/2, C0,i) at pressure PB2; their feeds being identical, their solutions are identical, and the system is solved once and scaled. Vessel V2C then receives their combined concentrate of flow rate 2Qr,V2A and composition Cr,V2A,i, at pressure PB2 − ΔPV2A, and the product is Qprod = 2Qp,V2A + Qp,V2C. In C3, the three vessels each receive (Q0/3, C0,i) at pressure PB2, are solved once and scaled by three; all concentrates are discarded, and the product is Qprod = 3Qp,V2A of composition Cp,V2A,i. In every case, the composition of a mixed stream is obtained from the species mass balance, Cmix,i = Σ Qk Ck,i/Σ Qk, applied to the streams being combined.

### 2.2. Solution–Diffusion Transport Model

Water and solute transport across each membrane element are described by the solution–diffusion framework [48]. Three ionic species representative of seawater are tracked: sodium (Na^+^), chloride (Cl^−^), and sulfate (SO_4_^2−^), each transported with an independent solute-permeability coefficient rather than through a lumped salinity, since it is the species-resolved permeate composition that determines compliance with the product specification. For each element, the governing relations solved simultaneously are the water-flux equation, the solute-flux equations for each species, the concentration-polarization relation, and the mass balances closing the feed, permeate, and reject streams:

Jw = Aw · (ΔP − Δπ)(1)

Js,i = Bi · (Cf,i · β − Cp,i)(2)

β = exp(0.7 · Qp/Qf)(3)

Qf = Qp + Qr(4)

Qf · Cf,i = Qp · Cp,i + Qr · Cr,i(5)

where Jw is the water flux, Aw is the water-permeability coefficient, ΔP is the transmembrane pressure, Δπ is the osmotic-pressure difference, Js,i and Bi are the solute flux and solute-permeability coefficient of species i, Cf,i, Cp,I, and Cr,i are the feed, permeate, and reject concentrations, β is the concentration-polarization factor, and Qf, Qp, and Qr are the feed, permeate, and reject volumetric flow rates. Equations (1) and (2) are the solution–diffusion relations [48]. The feed osmotic pressure is evaluated as π = 1.12·(273 + T)·Σmj (Equation (52) of the FilmTec Technical Manual [49]), and a temperature correction is applied through the temperature correction factor (TCF) of the same manual (its Equations (53) and (54)) [49]. Since all simulations are run at the standard reference temperature of T = 25 °C—the condition at which membrane manufacturers specify performance, enabling direct comparison with datasheet values and with the literature—the TCF equals unity and applies no correction; fixing this reference also isolates the effect of fouling from that of temperature variation. In Equation (3), the concentration-polarization factor follows the exponential form obtained from the FilmTec manual (its Equation (55)) [49]; it is an empirical closure of the film-theory description of concentration polarization, and the numerical coefficient is the manufacturer’s correlation. The factor is recomputed at every element and every instant from the local recovery, and therefore follows the drift of flux and recovery over the horizon; the argument is bounded to avoid numerical overflow at low feed flow, and that bound is shown in Section 2.7 to be inactive over the entire operating domain. Equations (4) and (5) state the element-wise continuity and species balances, following the element-by-element solution scheme used for spiral-wound systems [8,36]. The nonlinear system is solved element-by-element using the MATLAB fsolve routine, with the converged state of each element providing the feed condition for the next, which, for the three species tracked, amounts to ten simultaneous equations per element.

For the lead Pass-1 membrane (SW30HR-380), the water permeability is further corrected for operating pressure and feed flow rate through empirical factors fitted to the manufacturer projection software (WAVE), of the form Aw = Aw,0 · f(Pf) · g(Qf) · TCF · Ff, where f(Pf) is a linear decreasing function of feed pressure (capturing membrane compaction), g(Qf) is a quadratic function of feed flow (capturing the reduction in concentration polarization), and Ff = 0.85 is a membrane ageing factor. These correction factors were fitted by comparing the element-by-element SD model against WAVE projections, reducing the deviation to approximately 3%. Their product is verified in Section 2.7 to remain positive and bounded over the whole domain swept. The Pass-2 membranes use the nominal permeability without this correction.

### 2.3. Progressive Fouling Model

Progressive fouling is represented as a monotonic, time-dependent evolution of the two transport coefficients of the Pass-1 membrane over a 500-day horizon, discretized in 10-day steps (51 time points). Cake accumulation and surface coverage reduce the water permeability, while membrane degradation and the build-up of a solute-retaining fouling layer increase the solute permeability; in the base formulation, both effects are modelled as linear functions of normalized operating time:

A(t) = A0 · (1 − αA · t/500)(6)

Bi(t) = Bi,0 · (1 + αB · t/500)(7)

where A0 and Bi,0 are the initial water and solute permeabilities, t is the operating time in days, and αA and αB are dimensionless severity parameters; the same αB scales all three solute species.

The linear form of Equations (6) and (7) is adopted as a first-order representation of long-term permeability evolution, appropriate for the timescale and purpose of this study for three reasons. First, over the multi-year horizons relevant to design, the net drift of the transport coefficients—after the initial compaction transient and between cleaning cycles—is well approximated by a monotonic, near-linear trend, as reported in long-term field studies of full-scale RO operation [36,37]. Second, a linear law introduces only two interpretable parameters per coefficient, αA and αB, each with a direct physical meaning (the fractional change in permeability accumulated over the horizon) and each traceable to an independent field measurement, avoiding the over-parameterization of more elaborate fouling kinetics that cannot be constrained without plant-specific data. Third, because the central results concern the relative response of the architectures to the same imposed degradation, they depend on the severity and the coupling of the A(t) and B(t) trends rather than on the precise functional form. This last point is not assumed but tested: two alternative laws compiled in the review of Ruiz-García and co-workers [37] were also implemented, namely a power-law form of the type proposed by Wilf and co-workers, whose exponent was calibrated against permeate-flow declines of 20–25% in seawater plants, and the two-stage superposition of exponentials of Ruiz-García and Nuez [36], fitted to ten years of full-scale brackish-water operation, which separates an initial rapid stage attributed to compaction and irreversible fouling from a slower subsequent one. The three laws are calibrated to the same end-of-horizon state, so that all deliver identical transport coefficients at day 500; without that calibration, any difference between them would reflect the total degradation imposed rather than its distribution in time, whereas, as calibrated, the comparison isolates the trajectory. Results under the three laws are reported in the Supplementary Material and discussed in Section 4.5.

A single severity parameter αB is used for all three solutes on the basis of full-scale evidence: over 5026 days of brackish water reverse osmosis (BWRO) operation, Chebil et al. reported closely comparable increases in the ionic permeability coefficients of the main seawater ions (82% for the reference solute, and 88% and 87% for Cl^−^ and SO_4_^2−^) [50], so that a common multiplicative factor captures the measured behaviour without introducing species-specific parameters that the data do not warrant.

The parameter values defining the three fouling scenarios were fixed a priori from the literature on long-term RO performance decline. For the severe scenario, the water-permeability loss (αA = 0.25, i.e., a 25% decline in A) corresponds to the magnitude reported over extended operation of brackish-water FilmTec spiral-wound RO membranes by Abbas and Al-Bastaki [51], whereas the solute-permeability increase (αB = 0.82, i.e., an 82% rise in B) matches the average increase in B measured over the 5026 days of full-scale BWRO operation reported by Chebil et al. [50]. The low and moderate scenarios use proportionally smaller values, consistent with the predictive permeability-decline models reported by Ruiz-García and co-workers for long-term RO operation [36,37]. The 500-day horizon adopted here is that of the field study from which αA is taken [51], so that the severe scenario reproduces the reported decline over the period on which it was measured rather than extrapolating it, and for reference a full-scale seawater plant operated over four years reported a decline of about 30% in A together with an increase of about 70% in B [38]. These scenario parameters are listed together with the remaining operating conditions in Section 2.5.

Fouling is imposed on Pass 1 alone, and the justification for that boundary is the following. Fouling is conventionally classified as particulate and colloidal, organic, biological, and inorganic scaling. The first pass removes all particulate, colloidal, organic, and microbial material from the seawater feed, so that the second pass receives a stream in which the sources of the first three mechanisms are absent: there is no material available to form a cake layer, none to adsorb onto the membrane, and no microbial load to establish a biofilm. This last point extends beyond fouling itself, since the long-term rise in solute permeability of polyamide membranes is driven largely by biofilm-related effects and by the oxidative damage caused by the chemical cleanings that biofouling makes necessary; a pass that does not biofoul is not chemically cleaned. The fourth mechanism requires supersaturation, and here the model itself provides the quantitative argument: the permeate delivered by the first pass has a total dissolved solids content between 0.18 and 0.48 kg·m^−3^ across all architectures, scenarios and time points, against 33.6 kg·m^−3^ in the seawater feed, that is, between 70 and 184 times more dilute, and even the most concentrated stream anywhere in the second pass, its own concentrate at the end of the horizon under severe fouling, reaches at most 1.34 kg·m^−3^, still 25 times more dilute than the feed that the first pass processes continuously without scaling by design. These arguments are consistent with direct measurement, since membrane autopsies of a two-pass seawater train report significantly fewer deposited solids on the second-pass membranes than on those of the first [52]. The robustness of the results to this boundary is nevertheless tested by imposing degradation on the second pass as well, up to the extreme case in which it degrades as fast as the first; the analysis is reported in the Supplementary Material and discussed in Section 4.5.

### 2.4. Operating-Point Selection and Energy Metrics

At each time point and for each fouling scenario, the operating point is determined using a two-level procedure that minimizes specific energy consumption (SEC) while satisfying all technical and product-quality constraints. The product-quality constraint requires the final permeate concentration to remain at or below the PEM specification of 20 mg·L^−1^ (0.020 kg·m^−3^), which is the feed specification of the downstream polishing stage and not that of the electrolyzer, as established in the Introduction.

Pass 1. A feasibility scan is performed over a discrete grid of feed pressures (30–70 atm) and feed flow rates (3–14.1 m^3^·h^−1^). Each candidate is evaluated with the element-by-element vessel model and retained only if it satisfies the per-element and per-vessel pressure-drop limits, the maximum recovery, and the minimum-reject and maximum feed/permeate flow constraints. Feasible points are sorted by ascending Pass-1 SEC, and the first that also admits a feasible Pass-2 solution meeting the quality constraint is selected.

Pass 2. For the selected Pass-1 operating point, the feed pressure of the lead Pass-2 vessel is scanned over a fine grid (step 0.02 atm). In the three configurations, the operating point retained is the one yielding the global minimum Pass-2 SEC among all feasible pressures (rather than the first feasible point), so that the configurations are compared under an identical optimization criterion, within a single computational framework in which the selection rule, the fouling formulation, the constraint set, and the reporting are shared by construction.

The procedure minimizes the Pass-1 SEC and then, for the point so selected, the total SEC over the Pass-2 pressures, so that it is a sequential two-level optimization and not a joint minimization over both passes simultaneously; the point selected is therefore not necessarily the global minimizer of the total SEC. The distinction matters for how the absolute values are read and not for the comparison between architectures, which is the object of the study, since all three are evaluated under the identical rule. A joint optimization would require solving the second pass for every candidate first-pass point at every time step, which is computationally prohibitive at the temporal resolution used here, and the limitation is recorded in Section 4.9.

A physical-validity filter rejects any candidate whose converged permeate concentration or flow is non-physical (Cp ≤ 0, Qp ≤ 0, or non-finite), as a safeguard of the selection procedure.

The permeate flow rate is not a controlled set-point but an outcome of this procedure: as fouling reduces A(t), maintaining the product-quality constraint requires higher applied pressure, which raises both permeate flow and energy consumption. Pump power is evaluated as:

P = Pf · Q · kfac/ηp(8)

where Pf is the pump feed pressure (atm), Q the volumetric feed flow handled by the pump (m^3^·h^−1^), ηp = 0.85 the overall pump efficiency, and kfac = 101.325/3600 = 0.02815 kW·(atm·m^3^·h^−1^)^−1^ the unit-conversion factor from the pressure–flow product to electrical power.

The specific energy consumption (SEC) of the whole train is the sum of the two pump powers divided by the final permeate flow rate:

SEC = (PB1 + PB2)/Qp,final(9)

These definitions follow standard practice in the desalination energy literature [53]. Pressure losses within the membrane train are included in the model, since element-wise and vessel-wise pressure-drop correlations are applied at every element and enforced as operating constraints, so that the pressure available at each element, and hence the pump duty required, already accounts for the hydraulic losses along the pressure vessels. What lies outside the accounting is the balance of plant, as defined in the system boundary of Section 2, an exclusion consistent with the reported energy breakdown of reverse-osmosis systems, in which the high-pressure pump dominates the electrical demand of the membrane train, of the order of 70% of system energy use in the absence of energy recovery [54].

In the base case, no energy-recovery device is present. Where one is considered, a pressure-exchanger-type device transfers the hydraulic energy of the high-pressure Pass-1 concentrate to the incoming feed, so that the net electrical demand of the first-pass pump becomes:

PB1 = (Pf · Qf – ηERD · Pr · Qr) · kfac/ηp(10)

where Pr and Qr are the pressure and flow rate of the Pass-1 concentrate, and ηERD is the efficiency of the energy-recovery device. The device is integrated into the selection procedure rather than applied afterwards: the SEC of every candidate first-pass point is recomputed with recovery before the candidates are ranked, so that the point selected is the optimum of the system with recovery and not of the system without it. Recovery is applied to Pass 1 only, since the second pass operates between 3 and 10 atm and its concentrate carries no significant recoverable energy.

Three margin indicators are recorded at every time point in addition to the SEC. The quality margin is the difference between the product limit and the permeate concentration actually delivered at the selected point. The excess at the unconstrained optimum is the amount by which the permeate concentration at the energy-optimal point exceeds the limit, and is zero while that point remains usable and grows thereafter. The cost of quality compliance is the difference between the minimum total SEC with the product constraint imposed and the minimum without it, both evaluated over the same set of candidate points; because the constrained feasible set is a subset of the unconstrained one, this quantity is non-negative by construction, and it reports both when compliance begins to cost energy and how rapidly that cost accumulates.

### 2.5. Operating Conditions, Membrane Parameters and Constraints

The seawater feed composition and operating conditions are listed in Table 1; the progressive-fouling scenarios in Table 2; and the membrane transport coefficients, geometry, and technical limits in Table 3, with solute permeability resolved by species. Pressure-drop and pressure limits are specified in bar in the model and converted to atm (1 atm = 1.01325 bar). The first-pass operating grid is refined to the step sizes given in Table 1, for the reason established in Section 2.7, and no filtering of any kind is applied to the series reported in this work: all figures and statistics use the raw solver output.

**Table 2:** Progressive-fouling scenarios (αA, αB held constant over the horizon). The two right-hand columns give the resulting state of the first-pass membrane at the end of the horizon, which allows a plant to locate its own condition on this scale from measurements of normalized permeability and salt passage.

Scenario	αA	αB	Loss of A at 500 d	Increase in B at 500 d	Literature Basis
Low fouling (S1)	0.10	0.20	10%	20%	Conservative long-term decline
Moderate fouling (S2)	0.20	0.50	20%	50%	Intermediate, scaled per [36,37]
Severe fouling (S3)	0.25	0.82	25%	82%	αA per [51]; αB per [50]

Note: Time horizon = 500 days, Δt = 10 d (51 points). Severe-scenario coefficients from refs. [51] (αA) and [50] (αB); lower scenarios scaled per [36,37]. Fouling is not imposed on the second pass; the justification is given in Section 2.3.

**Table 3:** Membrane transport coefficients, geometry and technical limits. Solute permeability is resolved by species: the model transports sodium, chloride, and sulfate with independent coefficients rather than through a lumped salinity.

Property	SW30HR-380 (Pass 1)	XLE-440 (V2A, C1)	XLE PRO-4040 (Pass 2)
B_Na_ (m·s^−1^)	2.105 × 10^−8^	3.4105 × 10^−7^	3.0105 × 10^−7^
B_Cl_ (m·s^−1^)	2.305 × 10^−8^	3.490 × 10^−7^	3.100 × 10^−7^
B_SO4_ (m·s^−1^)	4.115 × 10^−9^	2.215 × 10^−7^	2.215 × 10^−7^
A (m·s^−1^·Pa^−1^	2.935 × 10^−7^	2.090 × 10^−6^	2.440 × 10^−6^
Element area (m^2^)	35.3	40.9	8.08
Elements per vessel	4	4	2
Max recovery (%)	10	30	30
Max feed pressure (atm)	81.9	40.5	40.5
Minimum concentrate flow (m^3^·h^−1^)	3.41	0.34	0.45
Maximum feed flow (m^3^·h^−1^)	14.1	17.0	3.2
Maximum permeate flow (m^3^·h^−1^)	1.14	1.94	0.38
Maximum element pressure drop (bar)	1.00	1.03	0.90
Maximum vessel pressure drop (bar)	3.50	3.45	2.07

Note: pressure-drop and pressure limits are specified in bar in the model and converted to atm (1 atm = 1.01325 bar). Values of A and B are given at 25 °C before the temperature and ageing corrections are applied.

### 2.6. Sensitivity Analysis

Because the fouling severity parameters αA and αB are fixed a priori rather than fitted to a specific plant, a joint sensitivity analysis was conducted to assess the robustness of the results to this choice. The severe-fouling scenario (αA = 0.25, αB = 0.82) was taken as the centre point of a two-level full factorial design, and the four corners of a ±25% perturbation box were evaluated for the two architectures of the controlled comparison. The perturbation magnitude of ±25% was selected to represent the uncertainty involved in transferring long-term fouling coefficients—originally measured on specific membranes and feedwaters [50,51]—to the configuration and conditions modelled here.

The factorial design was preferred over a denser grid for two reasons. The response is monotonic in both parameters, since more severe fouling always yields higher specific energy consumption, so that the envelope over the perturbation box is attained at its diagonal corners and interior points do not contribute to the uncertainty bands; it is the cross corners, where one parameter is high and the other low, that reveal interaction, whereas a denser grid adds resolution over a smooth surface but no further information about interaction. The three fouling scenarios of Table 2 already constitute a joint variation in much wider amplitude than ±25%, the two parameters rising by 150% and 310% simultaneously from the low to the severe scenario.

For each case, the full model was re-run over the 500-day horizon, and the end-of-horizon SEC was recorded together with the main effect of each parameter and the interaction term computed from the four corners. The diagonal corners provide the parametric envelope from which the uncertainty bands of the reported time series are derived. This parametric uncertainty is reported separately from the numerical uncertainty quantified in Section 2.7, since the two differ in magnitude by more than an order of magnitude and conflating them would misrepresent both. Results are reported and interpreted in Section 3.6 and Section 4.6.

### 2.7. Model Verification

The numerical core of the model was verified independently of its results, so that the numerical uncertainty is quantified rather than assumed. The complete set of checks is summarized in Table 4.

The first-pass operating point is selected from a discrete grid, and the first-pass energy objective is nearly flat near its minimum, so that the selected node changes as fouling advances and neighbouring nodes of almost identical first-pass energy deliver appreciably different flow and composition to the second pass. Refining the grid by a factor of 7.5 in the number of nodes changes the end-of-horizon specific energy consumption by 0.5% and reduces the amplitude of the resulting non-monotonic excursions by a factor of 5.4, from 0.19 to 0.04 kWh·m^−3^. The proportionality between refinement and residual identifies the excursions as a discretization artefact, and the residual, between 0.009 and 0.077 kWh·m^−3^ across the nine simulations, is adopted as the numerical uncertainty of the model.

Two further checks establish that the element-level solution is well posed. Re-solving the second-pass elements from an initial estimate adapted to their low-salinity feed reproduces the same root to within a relative difference of 7 × 10^−8^, so the solution does not depend on the starting point; over the admissible pressure range the computed permeate flow is linear in applied pressure, with successive increments of 0.119, 0.119 and 0.118 m^3^·h^−1^ per 2 atm, as the solution–diffusion formulation requires, so the solver reproduces the expected physical behaviour and not merely a converged number.

Two bounds present in the implementation were verified to be inactive over the whole operating domain and therefore incapable of influencing any result. The bound on the argument of the concentration-polarization expression, Equation (3), would require an element recovery of 429%, against manufacturer limits of 10% for the first-pass element and 30% for those of the second pass; the empirical permeability correction of Section 2.2 remains positive and bounded throughout, its product ranging between 0.773 and 1.225, with the vertex of the flow-dependent factor lying outside the range swept.

Over the nine simulations, the concentration-polarization factor ranges from 1.051 to 1.062, against a maximum admissible value of 1.073 imposed by the recovery limit of the first-pass element, so that the correction it applies to the osmotic pressure is between 5 and 6% and varies by about one percentage point over 500 days and across the full range of fouling severities examined. A recent comparison of polarization correlations for spacer-filled spiral-wound modules, based on computational fluid dynamics, reports mean discrepancies below 6% between the available correlations for the polarization modulus, against 83% for the pressure gradient [55]. A discrepancy of that order applied to a correction of that magnitude propagates to an effect on the reported specific energy consumption of the order of 0.3%, an order of magnitude below the numerical uncertainty quantified above and two orders below the parametric uncertainty of Section 2.6. However, the empirical closure adopted does not represent changes in channel hydrodynamics arising from the accumulation of a fouling layer, since fouling is represented here only through the evolution of the two transport coefficients; this is recorded among the limitations in Section 4.9.

## 3. Results

This section reports the simulation results for the two-pass SWRO train under three progressive-fouling scenarios (low, S1; moderate, S2; severe, S3) over a 500-day horizon, for the three candidate configurations. The three configurations share an identical first pass (two SW30HR-380 vessels in parallel, fed with standard seawater) and differ only in the Pass-2 arrangement: in C1, vessels V2A and V2B operate in series; in C2, V2A and V2B operate in parallel, and their combined concentrate feeds V2C in series; in C3, the three vessels operate in pure parallel. At each time step, the operating point minimizes specific energy consumption (SEC) subject to all technical constraints and the product-quality limit of 20 mg·L^−1^, under the single selection rule of Section 2.4.

Results are presented along the causal chain of the fouling process: the permeability decline, the hydraulic and quality performance that follows from it, the operating window and the energy cost of remaining within specification, the consolidated comparison between configurations, the effect of energy recovery, and the uncertainty of the results. Unless stated otherwise, the figures show the severe scenario, which is the condition under which the configurations differ most, and the three scenarios are reported together in Table 5. Four additional analyses—the three degradation laws, operation at fixed production, imposed second-pass degradation, and the attainable product quality at the energy optimum—are reported in the Supplementary Material and referred to from the subsections to which they belong. Interpretation and benchmarking against the literature are deferred to Section 4 (Discussion).

### 3.1. Membrane Permeability Degradation

**Figure 2:**
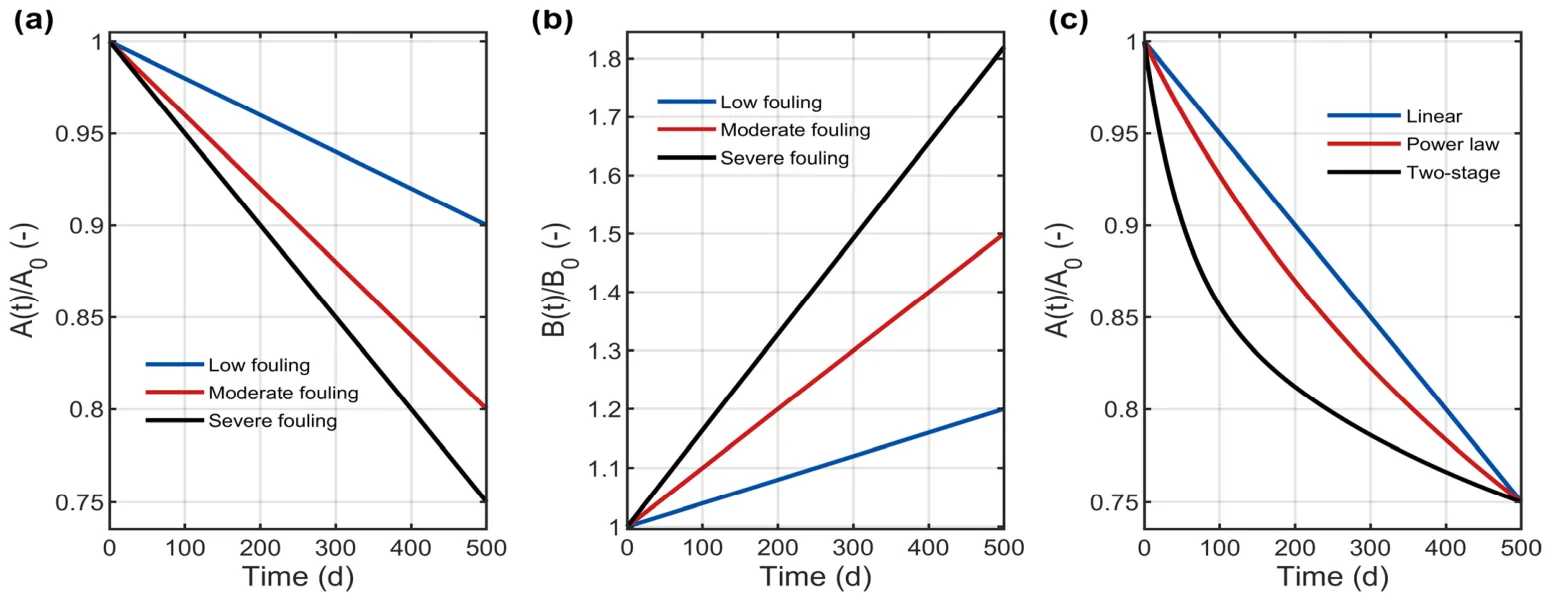
Normalized Pass-1 transport coefficients over the 500-day horizon for the three fouling scenarios: (**a**) hydraulic permeability A(t)/A0; (**b**) solute permeability B(t)/B0; (**c**) the three functional forms implemented for the decline of A under the severe scenario, calibrated to a common end-of-horizon state so that they differ in trajectory and not in accumulated magnitude. The curves are identical for the three configurations because fouling occurs during the shared first pass.

Progressive fouling is represented, as set out in Section 2.3, as a monotonic evolution of the two transport coefficients of the Pass-1 membrane, given by Equations (6) and (7), with the three scenarios differing only in the severity parameters αA and αB listed in Table 2. The fouling model therefore imposes a monotonic decline in the Pass-1 water permeability A(t), identical for the three configurations because fouling is applied to the shared first pass. Figure 2 shows the normalized permeability A(t)/A0. After 500 days, A(t)/A0 reaches 0.90, 0.80, and 0.75 for the low, moderate, and severe scenarios, respectively, reproducing the severity ordering defined by the fouling parameters. This decline is the physical driver of all subsequent operational effects. The normalized solute permeability B(t)/B0 rises over the same horizon to 1.20, 1.50, and 1.82 for the three scenarios. The severity levels are defined by the pair of parameters listed in Table 2 and are not inferred from an observable indicator; Table 2 additionally reports the end-of-horizon state each one produces, which allows a plant to place its own condition on that scale from measurements of normalized permeability and salt passage. The values of αA and αB are not assumed but taken from long-term field measurements, and the resulting performance decline is compared against reported plant data in Section 4.4.

Figure 2 also shows the three functional forms implemented for the decline of A, all calibrated to the same end-of-horizon state so that they differ in trajectory and not in accumulated magnitude. The linear and power-law forms are nearly indistinguishable at this degradation level, whereas the two-stage law concentrates the loss in the early horizon; the consequences of that difference are reported in the Supplementary Material and discussed in Section 4.5. Unless stated otherwise, the results that follow use the linear form. The restriction of fouling to the first pass, justified in Section 2.3, is confirmed quantitatively by the simulations themselves: across all configurations, scenarios and time points the permeate delivered by the first pass carry between 0.18 and 0.48 kg·m^−3^ of total dissolved solids, against 33.6 kg·m^−3^ in the seawater feed, and the most concentrated stream anywhere in the second pass reaches at most 1.34 kg·m^−3^.

### 3.2. Hydraulic and Quality Performance

**Figure 3:**
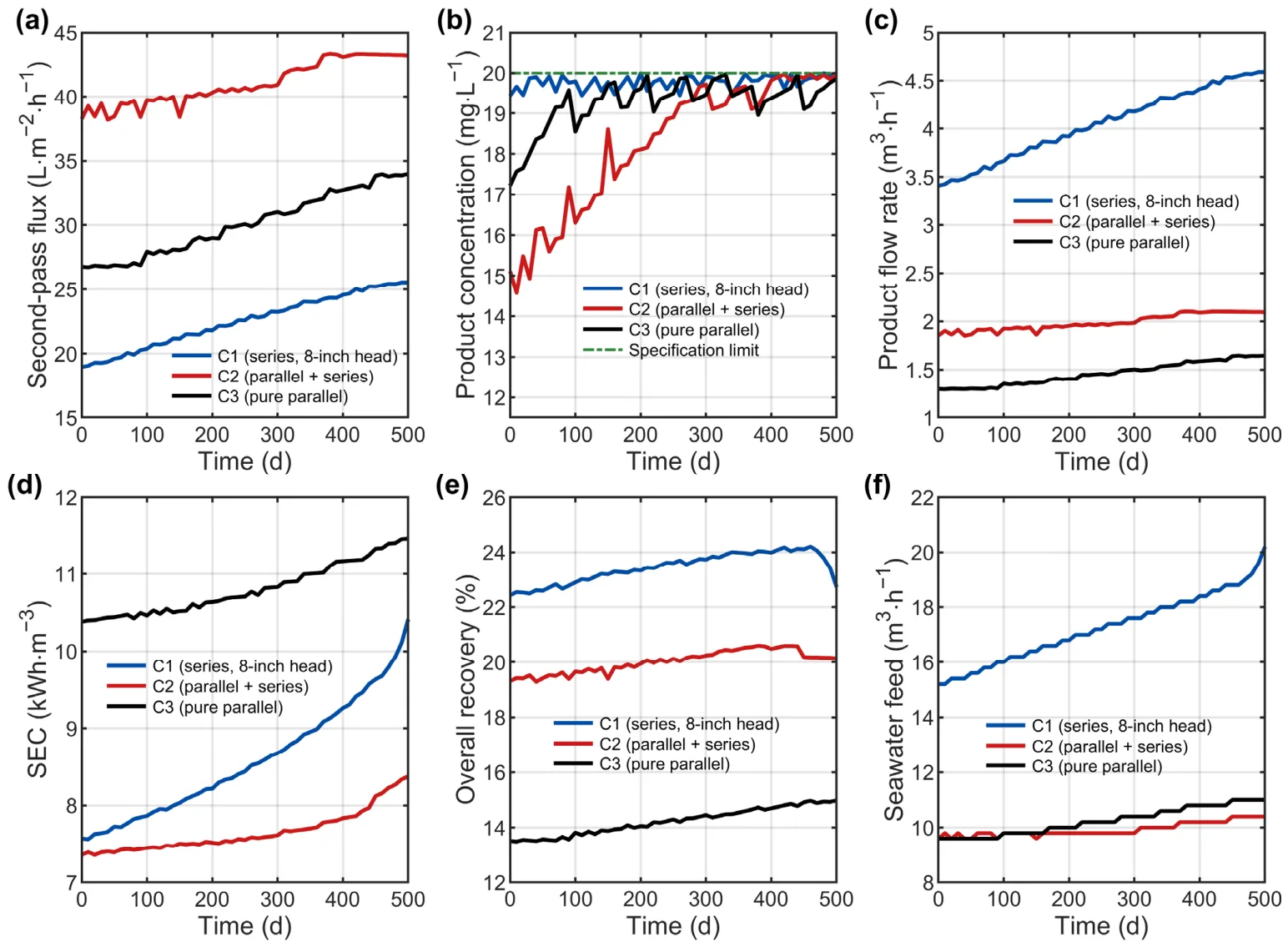
Performance of the three second-pass configurations over the 500-day horizon under severe fouling: (**a**) mean second-pass permeate flux; (**b**) product concentration against the 20 mg·L^−1^ specification; (**c**) product flow rate; (**d**) specific energy consumption of the train; (**e**) overall recovery referred to the total first-pass seawater feed; (**f**) seawater intake. The six panels describe a single behaviour: as the membranes degrade, the energy-optimal operating point moves towards higher pressure and higher output. All panels share a common vertical scale within each quantity.

Figure 3 reports the response of the three configurations to the imposed degradation, under the severe scenario. The six panels are read together, because they describe a single behaviour: as the membranes degrade, the operating point that minimizes the specific energy consumption of the train moves towards higher pressure and higher output, so that flux, product flow, product concentration, energy consumption, recovery and seawater intake all rise together. The system does not lose capacity as it fouls; it reallocates its operating point, and the cost of that reallocation is what the remainder of this section quantifies.

Panel (a) gives the mean second-pass permeate flux, which is the normalized quantity appropriate when configurations of different membrane areas are compared, and panel (c) gives the volumetric product flow. The contrast between the industrial reference and the two configurations of the controlled comparison is marked: C1 operates at 19–26 L·m^−2^·h^−1^ and C2 at 38–43 L·m^−2^·h^−1^, so that the hybrid arrangement sustains roughly twice the flux with 3.7 times less installed membrane area, while C3 lies between them at 27–34 L·m^−2^·h^−1^. In volumetric terms, the ordering reverses, with C1 delivering 3.4–4.6 m^3^·h^−1^ against 1.9–2.1 for C2 and 1.3–1.7 for C3, which reflects the difference in installed area rather than in performance. End-of-horizon values for the three scenarios are given in Table 5.

Panel (e) gives the overall recovery, defined as the final product flow relative to the total seawater feed of the first pass, that is, to the combined feed of both first-pass vessels, since the second pass receives the combined permeate of both. End-of-horizon values are 22.7–24.0% for C1, 20.0–20.7% for C2, and 14.0–15.0% for C3. C1 and C2 therefore achieve almost the same overall recovery, so the larger volumetric output of C1 arises from processing nearly twice the seawater feed and not from recovering a larger fraction of it, as panel (f) makes explicit: C1 draws 15.2–20.2 m^3^·h^−1^ over the horizon, against 9.6–10.4 for C2 and 9.6–11.0 for C3.

Because the permeate flow rate is an outcome of the optimization rather than a fixed set point, it does not collapse as permeability declines; instead, it drifts upward, slowly and monotonically, increasing over the horizon by 5.6–13.0% in C2 and by 5.8–27.0% in C3 depending on the fouling scenario. The direction deserves note, since a system losing water permeability might be expected to lose output: it does not, because as A declines, the energy-optimal operating point moves to higher pressure, and higher pressure yields more permeate. The consequences of instead holding production fixed are reported in the Supplementary Material and discussed in Section 4.5.

Product quality is the binding operational requirement: the permeate concentration must remain at or below 20 mg·L^−1^ at the inlet of the polishing stage. Panel (b) gives the product concentration against that specification. The three configurations start at different levels—approximately 19.5 mg·L^−1^ for C1, 15.1 for C2, and 17.2 for C3—and all three rise towards the limit as the membranes degrade. The rise has two causes acting together: the increase in the solute permeability imposed by Equation (6), and the displacement of the operating point towards higher output described above, which increases the solute load reaching the second pass. Quality is thus not merely eroded by fouling; part of the erosion is the price of the energy optimization itself, and this coupling is what Section 3.3 quantifies. All three configurations remain within specification throughout the horizon, and what varies is the energy cost of satisfying it.

The cost of sustaining product quality under fouling is energetic. Panel (d) gives the specific energy consumption of the train. C2 is the most efficient throughout, rising from 7.36 to 8.38 kWh·m^−3^ over the horizon; C1 rises from 7.57 to 10.42, overtaking C3 only in the final stretch; and C3 is the least efficient across almost the whole horizon, from 10.38 to 11.46. The two configurations of the controlled comparison differ by 36.9% at the end of the horizon under severe fouling, with membrane type, element count, and installed area held identical, so that the difference is attributable to the arrangement of the vessels alone. The corresponding figures for the three scenarios are consolidated in Table 5, and the mechanism is examined in Section 4.1.

### 3.3. Operating Window and the Cost of Quality Compliance

Section 3.2 established that the three configurations remain within specification throughout the horizon. That is a property of the selection rule, which cannot return a point violating the constraint, and it says nothing about how hard the constraint presses. This subsection reports what the constraint costs, and which constraint it is.

At every time point, the second-pass feed pressure was swept over its full admissible range 351 values, and the feasible pressures were counted twice: subject only to the manufacturer’s technical limits, and subject additionally to the product specification, the first count measuring hydraulic feasibility and the second separation feasibility. The counts are evaluated at the operating point that minimizes the first-pass specific energy consumption, and not at the point the model selects, because evaluated at the selected point there would be, by construction, at least one feasible pressure at every instant, the window could never close, and the quantity would be uninformative; evaluated at the energy-optimal point it reports how many pressures would be available if the system could operate there, and therefore when it ceases to be able to.

**Figure 4:**
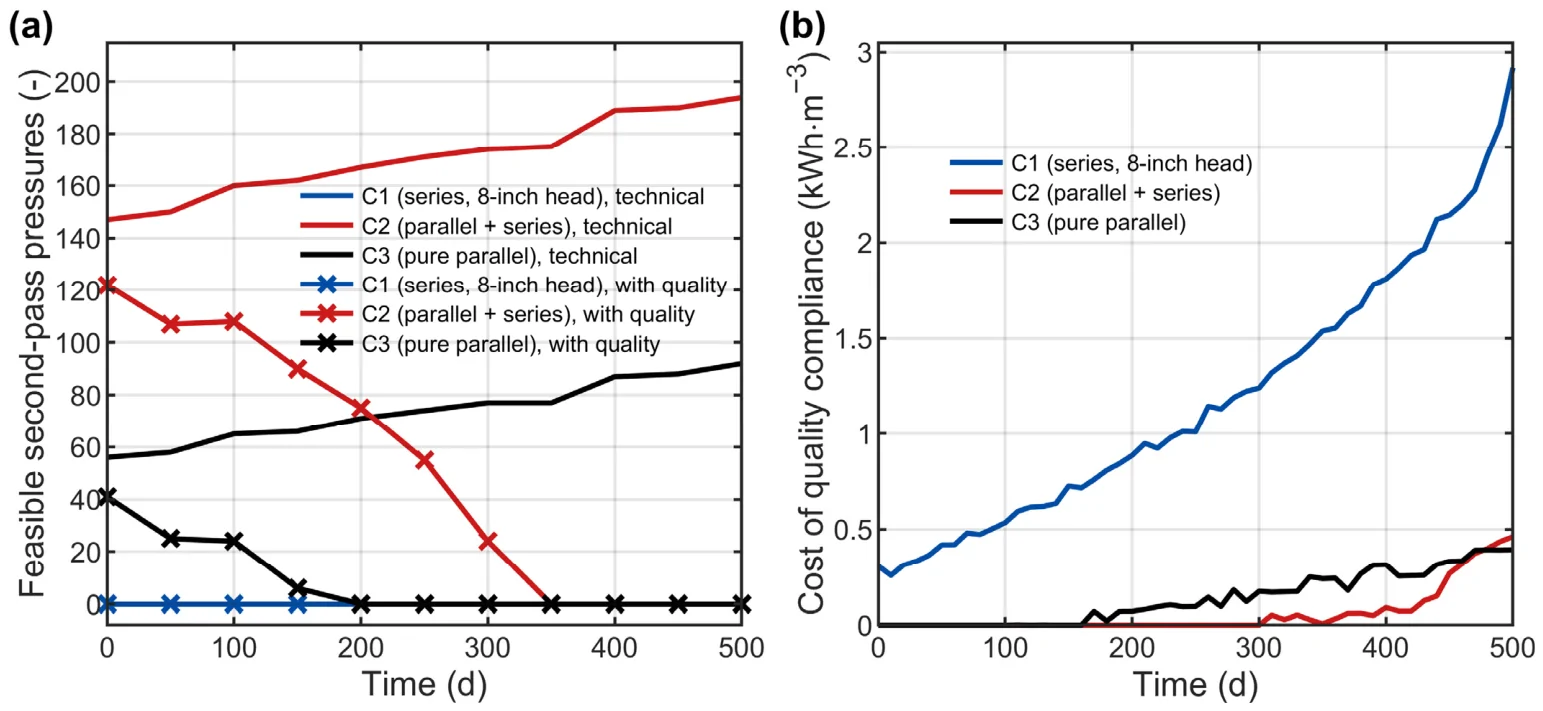
(**a**) Number of feasible second-pass feed pressures out of the 351 swept, counted subject only to the manufacturer’s technical limits and subject additionally to the product specification, under severe fouling. The counts are evaluated at the operating point that minimizes the first-pass specific energy consumption and not at the point the model selects; evaluated at the selected point, at least one feasible pressure would exist by construction at every instant, and the window could never close. For C1, both counts coincide at zero throughout the horizon, since C1 admits no feasible second-pass pressure at the first-pass energy optimum (Section 3.3); its two series therefore overlap at the bottom of the panel. (**b**) Cost of quality compliance over the horizon, defined as the difference between the minimum total specific energy consumption with and without the product constraint imposed.

Figure 4a gives both counts over the horizon under severe fouling, and the result is unambiguous. The technically feasible window does not contract as the membranes degrade: it widens, from 147 to 194 admissible pressures in C2 and from 56 to 92 in C3. The window that also satisfies the product specification contracts monotonically and closes between days 300 and 350 in C2 and between days 150 and 200 in C3. Progressive fouling therefore constrains this system through separation and not through hydraulics, and the two configurations of the controlled comparison exhibit the behaviour independently. Tabulated counts are given in Table S4.

The industrial reference behaves differently, and the difference is instructive. C1 admits no feasible second-pass pressure at the first-pass energy optimum at any time point, including day zero, because its head vessel, an 8-inch XLE-440 element, exceeds its recovery limit at every admissible pressure when fed with the permeate that the first pass delivers at that point: the element is oversized relative to that flow. C1 therefore never operates at the first-pass energy optimum and must instead descend the ordered list of feasible points, forcing the first pass to overproduce in order to supply its second pass. This is a capacity limitation and not a separation one; it is the mechanism behind the intake and flux figures of Section 3.2, and its consequences for design are taken up in Section 4.1.

The closure of the quality window has a direct reading in concentration. Table S5 gives, at the first-pass energy optimum and with the quality constraint lifted, the best product concentration each configuration could attain: it crosses the 20 mg·L^−1^ specification between days 150 and 200 in C3 and between days 300 and 350 in C2, precisely when the corresponding window closes, and from those instants onward the system must abandon the first-pass energy optimum in order to meet the specification.

That abandonment has a price, quantified by the cost of quality compliance defined in Section 2.4 and shown in Figure 4b. C1 pays this cost from day zero in all three scenarios, which is the energetic expression of the capacity limitation described above, and it reaches 2.92 kWh·m^−3^ by the end of the horizon under severe fouling. C2 pays nothing at all in the low and moderate scenarios, and only from day 310 under severe fouling, ending at 0.46 kWh·m^−3^. C3 begins to pay at day 320 under moderate fouling and at day 170 under severe fouling, ending at 0.39 kWh·m^−3^. Onset therefore favours C2, which sustains its unconstrained optimum about 1.8 times longer than C3 under severe fouling, whereas the subsequent rate does not: once each configuration has departed from its optimum, the compliance cost accumulates about twice as fast in C2 as in C3. The two facts are reported together because reporting only the first would misrepresent the comparison, and the mechanism behind the asymmetry is examined in Section 4.1.

### 3.4. Consolidated Comparison

The operational contrast between the configurations is summarized in Table 5, which consolidates the key performance metrics at the beginning and at the end of the horizon across the fouling scenarios, reporting specific energy consumption and its increase, product flow, overall recovery, second-pass flux, seawater intake, and the cost of quality compliance with the day on which it first becomes non-zero.

With membrane type, element count, and installed area held identical, the arrangement of the second-pass vessels alone changes the end-of-horizon specific energy consumption by 40.8%, 41.6%, and 36.9% in the low, moderate, and severe scenarios: C3 consumes that much more than C2 in every case. The ordering is preserved across the three scenarios, and the gap is of the same order in all of them; since every other design variable is fixed, the difference is attributable to the topology and to nothing else.

The configurations differ not only in level but in how much fouling costs them. From the low to the severe scenario the end-of-horizon specific energy consumption rises by 11.6% in C2 and by 8.5% in C3, against 29.7% in C1; within each configuration the rise over the 500-day horizon is likewise modest for the two of the controlled comparison, 2.0% to 13.8% in C2 and 1.7% to 10.4% in C3, and considerably larger for the industrial reference, 6.1% to 37.6%. C1 starts at 7.57 kWh·m^−3^, close to C2, and ends at 10.42 under severe fouling, overtaking C3 only in the final stretch of the horizon while drawing approximately twice the seawater and installing 3.7 times the membrane area of either, with a cost of quality compliance non-zero from day zero in all three scenarios for the capacity reason given in Section 3.3.

### 3.5. Energy Recovery

The results reported so far correspond to a train without an energy-recovery device. This subsection reports the effect of adding one, with the device integrated into the selection of the operating point as described in Section 2.4, so that the specific energy consumption of every candidate first-pass point is recomputed with recovery before the candidates are ranked and the point selected is the optimum of the system with recovery.

Table 6 gives the end-of-horizon values under severe fouling at a device efficiency of 0.96. The specific energy consumption falls from 10.415 to 3.796 kWh·m^−3^ in C1, from 8.376 to 2.999 in C2, and from 11.464 to 4.217 in C3, that is, reductions of 63.6%, 64.2%, and 63.2%. The three are nearly identical, as expected, since the device acts on the first pass, which is common to the three configurations. C2 remains the most efficient and C3 the least, with or without recovery, and the gap between the two configurations of the controlled comparison is 36.9% without recovery and 40.6% with it, so that the comparison between second-pass arrangements does not depend on the presence of the device. This is not a trivial consequence of applying the same device to a common first pass, since recovery favours C1 disproportionately, C1 rejecting approximately twice the concentrate from which energy is recovered, and C1 and C2 differ by only 0.21 kWh·m^−3^ at the start of the horizon.

**Figure 5:**
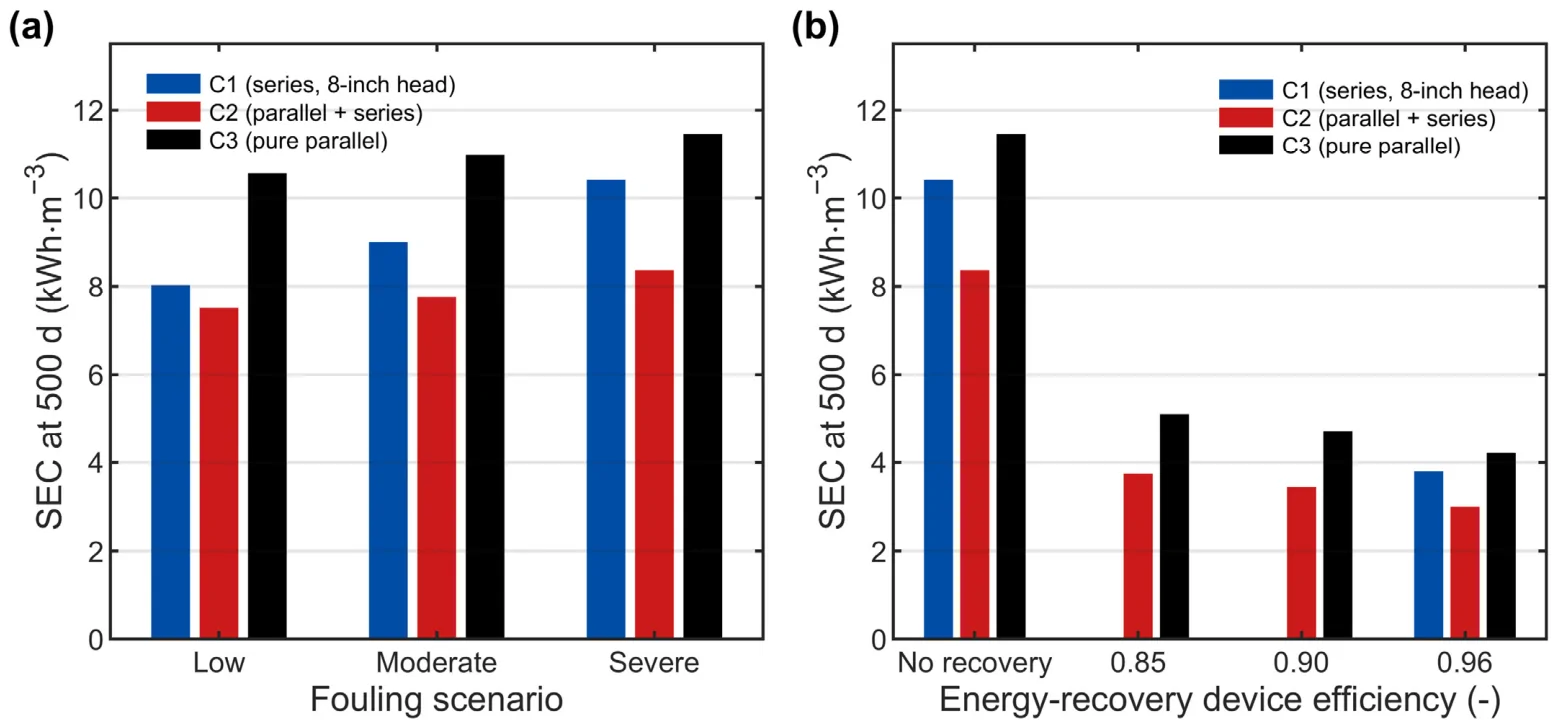
Energy performance. (**a**) End-of-horizon specific energy consumption of the three configurations as a function of fouling severity; (**b**) effect of the energy-recovery device on the specific energy consumption at 500 d, at device efficiencies spanning the range of commercial technologies. In panel (**b**), the conventional configuration C1 was evaluated only without recovery and at an efficiency of 0.96; the intermediate efficiencies were computed for the two configurations, controlled for membrane type, element count, and installed area, for which the comparison is strict. The absent bars therefore indicate cases not evaluated, not cases without a solution.

Implementing the efficiency curve of a specific device requires manufacturer data for that model. What can be established, and answers the underlying question, is whether the results depend on the efficiency value within the range spanned by commercial technologies. Reported efficiencies, defined as the change in feed pressure divided by the change in concentrate pressure, are 75% for turbines, 80% for turbochargers, and 85% for Pelton wheels, while isobaric chambers reach 95–97% [56]; pressure exchangers are reported to recover up to 98% of the pressure energy of the brine, and Pelton turbines and turbochargers around 90% [57]. The analysis was accordingly repeated at efficiencies of 0.85 and 0.90, which together with the cases at 0 and 0.96 span that range, and the results are given in Figure 5b and in Table 7. The gap between C2 and C3 is 36.9% without recovery, 36.3% at 0.85, 37.1% at 0.90, and 40.6% at 0.96, a variation of 4.3 percentage points across the entire commercial range, against 27.8% to 42.5% for the parametric uncertainty of the fouling coefficients reported in Section 3.6. The efficiency of the device therefore matters considerably less to the comparison between configurations than the values of the fouling parameters, and representing the device by a constant efficiency is adequate for that comparison.

Two further effects appear once the device is integrated. Without recovery, the specific energy consumption of C2 rises by 1.02 kWh·m^−3^ over the horizon under severe fouling, and with recovery by 0.53 kWh·m^−3^, so that approximately half of the energy cost of progressive fouling is absorbed by the device, because a substantial part of the additional pressure required is recovered from the concentrate rather than dissipated. The corresponding time series, with and without recovery, are given in Figure S5. And because recovery returns most of the pressurization energy, the penalty for operating at low recovery largely disappears and the energy optimum moves towards lower first-pass pressure and lower recovery, which yields a more concentrated first-pass permeate: under severe fouling the unconstrained energy optimum would deliver a product of 35.5 mg·L^−1^ at the start of the horizon and 64.1 mg·L^−1^ at its end in C2, against 61.8 mg·L^−1^ in C1 and 34.7 mg·L^−1^ in C3, all against a specification of 20 mg·L^−1^. The system therefore operates permanently away from its energy optimum in order to meet the specification; the distance grows as the membranes degrade, and the effect scales with the efficiency of the device, since the mean distance of the selected operating point from the unconstrained optimum grows monotonically from the case without recovery through efficiencies of 0.85 and 0.90 to 0.96. Even so, the energy saving remains of the order of 64%. The implication for design is taken up in Section 4.3.

### 3.6. Sensitivity to the Fouling Severity Parameters

**Figure 6:**
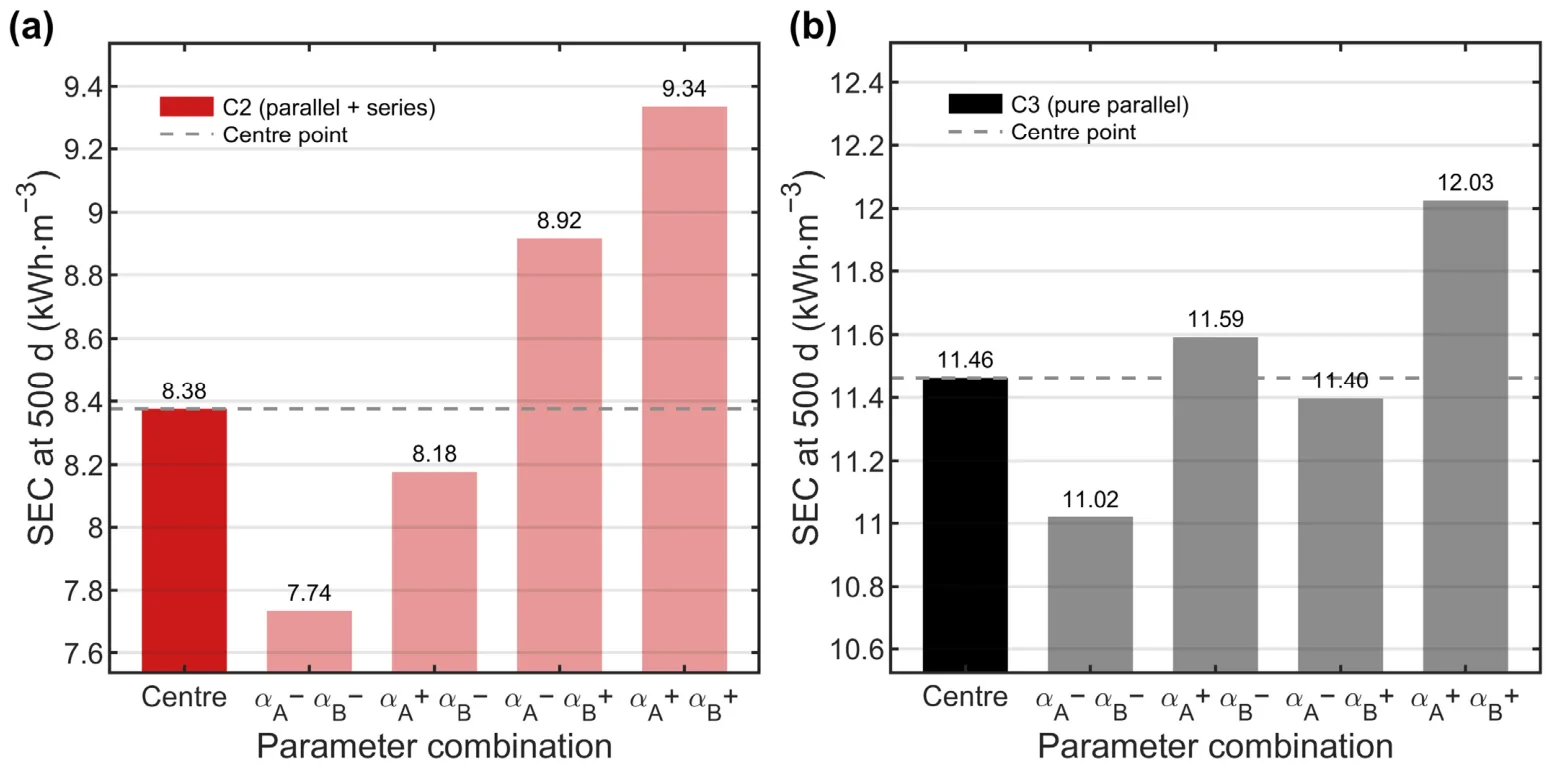
End-of-horizon specific energy consumption at the five combinations of the factorial design, for (**a**) configuration C2 and (**b**) configuration C3. The dashed line marks the centre point, the severe-fouling base case. The ordering of the configurations is preserved in every combination, while the magnitude of the gap varies between 27.8% and 42.5%.

The results presented in Section 3.1, Section 3.2, Section 3.3, Section 3.4 and Section 3.5 correspond to fixed values of the fouling severity parameters (αA, αB). To assess the robustness of these results to that a priori choice, a joint sensitivity analysis was performed around the severe-fouling base case (αA = 0.25, αB = 0.82) using the two-level full factorial design of Section 2.6, evaluated for the two configurations of the controlled comparison. The end-of-horizon SEC was recorded for each case, together with the main effect of each parameter and the interaction term computed from the four corners; results are summarized in Table 8 and Figure 6.

The interaction term is −0.012 kWh·m^−3^ in C2 and 0.029 in C3, that is, 1.0% and 4.9% of the corresponding main effects, so that over the range examined the two fouling parameters act independently and their effects combine additively; the response can therefore be described by its main effects without a cross term.

Each configuration is dominated by a different parameter. In C2, the solute-permeability parameter dominates, with a main effect of 1.171 kWh·m^−3^ against 0.430 for the water-permeability parameter; in C3, the ordering reverses, with 0.404 against 0.599. The two configurations are thus vulnerable to different degradation mechanisms, which is consistent with the behaviour reported in Section 3.3: C2 operates against the product specification, so degradation of salt rejection affects it directly, whereas C3 divides the feed among three branches, operates each vessel at lower recovery and retains more quality margin, so that what limits it is the loss of water permeability. The implication for membrane selection and monitoring is taken up in Section 4.6.

**Figure 7:**
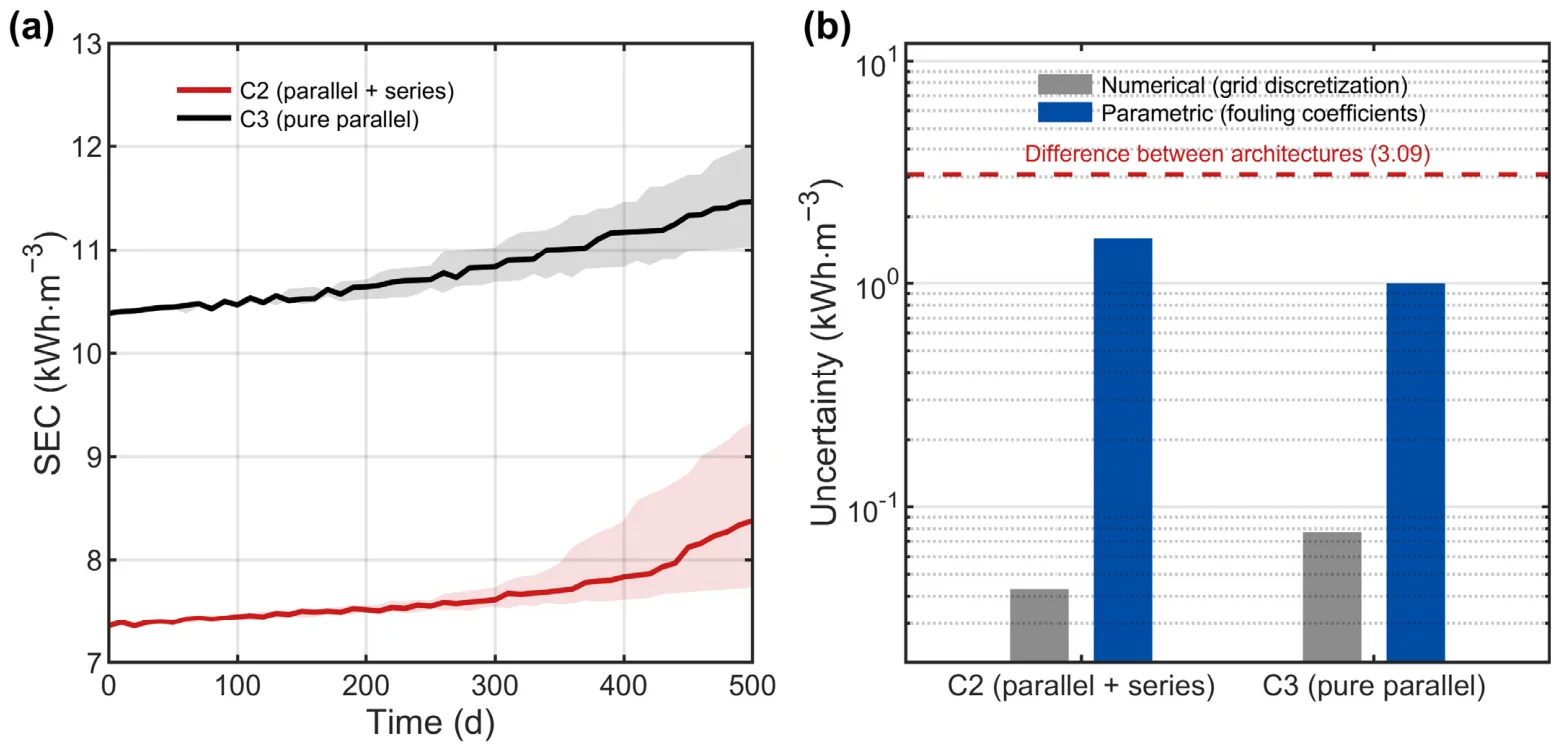
(**a**) Specific energy consumption over the horizon under severe fouling, with bands derived from the diagonal corners of the factorial perturbation box. (**b**) Numerical and parametric uncertainty compared on a logarithmic scale, against the difference between the two configurations at the end of the horizon. The parametric uncertainty exceeds the numerical one by more than an order of magnitude, and both remain below the difference the model is used to resolve.

The ordering of the configurations is preserved in all five parameter combinations, so the qualitative conclusion does not depend on the exact values of the fouling parameters. The magnitude of the gap ranges from 27.8% to 42.5% across the perturbation box, against 36.9% at the centre point, and both facts are reported since presenting the gap as a single figure would overstate the precision available. Because the response is monotonic in both parameters, the envelope over the perturbation box is attained at its diagonal corners, and those corners provide the bands shown around the time series in Figure 7a; the end-of-horizon band width is 1.601 kWh·m^−3^ in C2 and 1.003 in C3, that is, 19.1% and 8.7% of the respective central values. The asymmetry is itself informative, since C2 is the more sensitive of the two because it is dominated by the solute-permeability parameter, which carries the wider relative perturbation.

Figure 7b compares the two sources of uncertainty on a logarithmic scale. The numerical uncertainty, arising from the discretization of the operating grid and quantified in Section 2.7, is 0.04 to 0.08 kWh·m^−3^, whereas the parametric uncertainty, arising from the fouling coefficients, is 1.00 to 1.60 kWh·m^−3^. The dominant source of uncertainty in this model is therefore the value of the fouling parameters and not the numerics, by more than an order of magnitude, and both remain below the difference between the configurations, which is 3.09 kWh·m^−3^ at the end of the horizon under severe fouling, so that the comparison is resolved by the model with margin.

## 4. Discussion

### 4.1. Mechanistic Origin of the Energy Difference Between Configurations

The dynamic simulation of the two-pass SWRO train under progressive fouling reveals a clear and consistent pattern: the three candidate configurations are not merely points on a common efficiency curve, but respond to the same imposed degradation in structurally different ways. Under clean-membrane conditions, C1 and C2 are nearly equivalent in specific energy consumption, 7.57 and 7.36 kWh·m^−3^, while C3 stands well above them at 10.38. As fouling progresses, their behaviour diverges: C1 rises monotonically with severity, from 8.03 kWh·m^−3^ under mild fouling to 10.42 under the severe scenario, whereas C2 and C3 rise far more moderately, by 11.6% and 8.5%, respectively, over the same range of severities.

The divergence stems from how each configuration must respond, through the optimizer, to the progressive loss of water permeability. As the water-permeability coefficient A declines with fouling, maintaining the product specification requires raising the Pass-1 feed pressure, and because pump power scales with the product of pressure and flow, the magnitude of that compensating response governs the energy trajectory. This is the mechanism by which fouling raises the specific energy consumption of reverse-osmosis systems generally [29]. What differs between the configurations is how much pressure escalation each one requires, and the controlled comparison shows that the vessel arrangement alone determines it.

Configurations C2 and C3 employ the same membrane element, the same number of elements, and the same installed area, and differ by 36.9% to 41.6% in specific energy consumption depending on the scenario. The hybrid arrangement of C2 places a vessel in series behind the parallel pair, which receives the combined concentrate of the two upstream vessels at their residual pressure and recovers additional permeate from it without further pumping, whereas the pure-parallel arrangement of C3 has no equivalent stage: its three vessels each receive one third of the Pass-1 permeate and their concentrates are discarded at whatever pressure they retain. The consequence is the recovery reported in Section 3.2, 20.0–20.7% in C2 against 14.0–15.0% in C3 for essentially the same seawater intake. Both configurations pressurize a comparable feed; one extracts more product from it, and since the specific energy consumption is pump power divided by product flow, that difference in recovery is the difference in energy per unit of product [58].

The direction of the effect requires qualification, because adding stages does not improve efficiency unconditionally: superstructure-based optimization finds that increasing the number of stages can raise the specific energy consumption, each additional stage operating on a more concentrated and less permeable stream and possibly requiring its own pressurization [42]. The present case avoids that penalty for a specific reason, namely that the series vessel of C2 is driven by residual pressure alone and adds no pump duty, so the permeate it recovers is obtained at essentially zero marginal energy. Where a series stage requires interstage pressurization, the balance would differ, and the result should not be extrapolated to that case.

Dividing the feed among more branches has a second consequence that reinforces the first. Each vessel of C3 operates at a lower feed flow and therefore reaches its recovery limit at a lower pressure, which narrows the range of admissible operating pressures: 56 to 92 feasible pressures in C3 against 147 to 194 in C2. That the arrangement of the vessels governs the extent of the feasible region, and with it the position of the minimum-energy point, is consistent with the operating-window analyses of alternative vessel configurations of Ruiz-García and co-workers [41], and a narrower admissible range affords fewer opportunities to find a point that is simultaneously efficient and compliant, so that the energy penalty of the product constraint is correspondingly larger. The advantage of the hybrid arrangement is thus traceable to a specific structural feature rather than to topology in the abstract, which identifies the condition under which it would be lost: the series vessel operates on the most concentrated stream of the second pass and is the element most exposed to any degradation occurring there, and Section 4.5 reports the test of that prediction.

The industrial reference is limited by a different mechanism, and the constraint diagnosis of Section 3.3 shows why it behaves as it does. Its head vessel carries an 8-inch XLE-440 element of 40.9 m^2^ active area, against 8.08 m^2^ for the 4-inch elements of the other configurations, and fed with the permeate that the first pass delivers at its energy optimum, that element exceeds its recovery limit at every admissible pressure, from the first day and in every scenario. The limitation is one of capacity rather than of separation, and its consequences propagate upstream: unable to operate at the first-pass energy optimum, C1 must descend the ordered list of feasible points until it reaches one whose permeate flow keeps its head vessel below the recovery limit, which means forcing the first pass to overproduce. It draws 15.2 to 20.2 m^3^·h^−1^ of seawater against 9.6 to 10.4 in C2, delivering roughly twice the product at 59% of the flux, with a cost of quality compliance non-zero from day zero that reaches 2.92 kWh·m^−3^ under severe fouling.

This interpretation is consistent with prior work showing that the position of a spiral-wound module within a pressure vessel governs how strongly a given permeability loss propagates into the system-level energy demand [8], and with the general observation that fouling raises SEC primarily through the increased feed pressure required to maintain flux [29]. Whereas those studies resolve the fouling–pressure–energy mechanism within a single vessel or a fixed layout, the present analysis resolves it at the level of complete second-pass configurations evaluated under identical feed and fouling conditions and, for two of them, at identical membrane type and installed area; the present result also extends the earlier observation across passes, since here the mismatch of a single second-pass element displaces the operating point of the first. The practical consequence is that the architecture of the desalination train—not only its membranes or operating point—becomes a design lever for the energy performance of a desalination–electrolysis system under realistic, time-evolving fouling. The corresponding implication for sizing is direct: the second-pass element should be dimensioned against the permeate flow that the first pass delivers at its own optimum, and not by analogy with first-pass practice or by nominal capacity, since where the two are mismatched the penalty is paid upstream, in intake and pumping power, and from the first day of operation. This is consistent with the modular logic under which reverse-osmosis capacity is designed [20,21]: the train must be internally consistent before it is replicated.

### 4.2. Separation-Limited Operation Under Progressive Fouling

The most operationally consequential result of this work concerns not how much energy each configuration consumes but which constraint eventually limits it. The framing implicit in much of the fouling literature is hydraulic: as the membranes foul, the water permeability falls, the pressure required rises, and the system approaches its pressure and recovery limits. Section 3.3 shows that in this system the sequence does not occur.

The technically feasible window of second-pass operating pressures does not contract as the membranes degrade. It widens, from 147 to 194 admissible pressures in C2 and from 56 to 92 in C3 over the 500-day horizon, whereas the window that also satisfies the product specification contracts monotonically over the same period and closes between days 300 and 350 in C2 and between days 150 and 200 in C3. Two configurations that differ only in the arrangement of their vessels exhibit the behaviour independently, and the corresponding attainable product quality at the energy optimum crosses the 20 mg·L^−1^ specification at precisely those instants. Progressive fouling therefore constrains this system through separation and not through hydraulics.

The mechanism is intelligible once the two effects of fouling are separated. The decline of the water-permeability coefficient A and the rise in the solute-permeability coefficient B act on the system in opposite directions with respect to feasibility: a falling A requires higher pressure to sustain a given flow, which consumes energy but enlarges the admissible pressure range, since the vessel reaches its recovery limit later, so that the window widens for the same reason that the energy cost rises; whereas a rising B degrades rejection at every pressure and shifts the attainable product concentration upward, which contracts the range of pressures compatible with the specification. The coupling explains why the sensitivity analysis of Section 3.6 finds C2 dominated by αB and C3 by αA, since C2 operates against the quality constraint and is therefore governed by the parameter that erodes rejection, whereas C3, dividing the feed among three branches and operating each vessel at lower recovery, retains more quality margin and is governed by the parameter that erodes permeability.

This result does not overturn the established understanding that fouling raises energy demand through the pressure required to maintain flux [29]; that mechanism operates here and is what Section 4.1 describes. What it adds is that the pressure escalation is not what eventually stops the system: within the range of degradation examined, and within manufacturer limits, the hydraulic response has room to spare while the separation performance does not. The distinction matters because the two failure modes call for different responses: a hydraulically limited system being relieved by additional pressure capacity or by reducing flux, whereas a separation-limited one is relieved only by restoring rejection or by adding membrane area.

Because no configuration exhausts its admissible operating set within the horizon, the relevant question is not when operation becomes impossible but when it ceases to be free, and the cost of quality compliance measures exactly that: it is zero while the energy optimum remains within specification and positive thereafter, and it is non-negative by construction. Under severe fouling, C2 sustains its unconstrained optimum until day 310 and C3 only until day 170, so onset favours the hybrid arrangement by a factor of about 1.8; yet the subsequent rate reverses the ordering, since once each has departed from its optimum the compliance cost accumulates about twice as fast in C2 as in C3. Both facts are reported because they answer different operational questions: the first, how long the plant runs at its design efficiency, and the second, how quickly efficiency deteriorates once it does not. The asymmetry follows from the same structural feature identified in Section 4.1: the series vessel of C2 recovers permeate from the most concentrated stream of the second pass, and it is that stream whose quality degrades fastest as B rises, so the configuration whose advantage depends on the most concentrated stream is the one whose advantage that degradation erodes most rapidly, while C3, having no such stage, has correspondingly less to lose.

The dynamic model provides a means of anticipating these instants at the design stage rather than encountering them in operation, and of bounding their uncertainty, since Section 3.6 shows that the onset day is governed primarily by αB in C2, so that a plant able to monitor the rise in its salt passage can update the estimate as it operates. For the design of an integrated desalination–electrolysis facility the practical reading follows: where continuity of hydrogen production at design efficiency is paramount, the arrangement that sustains its unconstrained optimum longest is preferable and cleaning should be scheduled before that onset rather than in response to a rising energy bill; where maintenance intervals are fixed by other considerations, the relevant criterion is instead the rate at which the compliance cost accumulates within the interval. The two criteria do not select the same configuration, and the choice between them is a plant-level decision rather than a membrane-level one.

### 4.3. Energy Consumption in Context: The Role of Energy Recovery

The absolute SEC values obtained in this study (7.4–11.5 kWh·m^−3^ without energy recovery) are higher than those commonly cited for commercial SWRO, which typically fall between 2.5 and 4.0 kWh·m^−3^ for the RO stage and 3.5–5.0 kWh·m^−3^ at plant level when pre- and post-treatment are included [16,59]. This difference is expected and is primarily due to a deliberate modelling choice: the base case does not include an energy recovery device (ERD). The relevance of this distinction is well documented. Zhang et al. report that, for reverse-osmosis desalination without any energy-recovery equipment, the high-pressure pump alone accounts for roughly 70% of system energy use and the consumption is on the order of 8–10 kWh·m^−3^, falling to 3–4.5 kWh·m^−3^ once high-pressure brine energy is recovered [54]. Our values therefore sit squarely within the range reported for no-ERD systems, rather than being anomalous. For additional context, the practical thermodynamic limit of SWRO is about 1.6 kWh·m^−3^ at 35 g·L^−1^ and 50% recovery, and single-stage units with energy recovery operate below 3 kWh·m^−3^ [60]; historically, SWRO has fallen from roughly 15 kWh·m^−3^ in the 1970s to under 2 kWh·m^−3^ today, a reduction attributable largely to improved membranes, higher-efficiency pumps and the adoption of ERD [61]. A second contribution to the difference is intrinsic to the system modelled, since a two-pass train delivering water to a polishing stage carries the energy demand of a second pressurization that single-pass plants producing potable water do not.

To quantify how much of the gap is attributable to the absence of an ERD, the device was integrated into the selection of the operating point according to Equation (10) rather than applied as a post-calculation, as reported in Section 3.5. At a device efficiency of 0.96, the end-of-horizon values under severe fouling fall from 10.415 to 3.796 kWh·m^−3^ in C1, from 8.376 to 2.999 in C2 and from 11.464 to 4.217 in C3, reductions of 63.6%, 64.2% and 63.2%. With recovery, the values approach, without reaching, those of single-pass commercial plants, which is consistent with the additional demand imposed by a second pass and by a product specification set by a polishing stage rather than by potable-water quality.

Two features of this calculation deserve emphasis. The first is that the device must be integrated into the operating-point selection rather than applied afterwards, since holding the operating point fixed and subtracting the recovered energy cannot represent the reallocation that recovery induces, and that reallocation is substantial: Fang reports that improving the efficiency of the recovery device shifts the optimal recovery of a reverse-osmosis system from 45.1% to 25.3% [62]. The second is that the magnitude obtained lies at the upper end of what operating plants report, since full-scale facilities report energy savings of 35–42% with Pelton turbines and 55–60% with isobaric systems [63], and an exergy analysis of an operating two-pass RO unit, modelled and validated against plant data, reports reductions of about 30% in total power consumption with a recovery turbine and about 50% with a pressure exchanger [64]. The value calculated here at efficiencies of 0.85 to 0.90 is 55–59%, within that range, whereas the 64% obtained at 0.96 lies above it, consistent with an efficiency at the upper end of the commercial range and with a system boundary that excludes intake, pretreatment and balance of plant.

Two points deserve emphasis. First, even with energy recovery, the estimated SEC remains above that of single-pass commercial plants, which is consistent with the additional demand imposed by a second pass and by the product quality targeted here, rather than potable-water quality. Second, and most importantly, the comparative ranking is preserved: C2 remains more efficient than C3 under every scenario, with or without energy recovery, and the gap between them is 36.9% without recovery and 40.6% with it. Across the range of efficiencies spanned by commercial technologies, from 75–85% for centrifugal devices to 95–97% for isobaric chambers [56,65], the gap varies by 4.3 percentage points, against 27.8% to 42.5% for the parametric uncertainty of the fouling coefficients, so that the efficiency of the recovery device matters considerably less to the comparison than the values of the fouling parameters and representing the device by a constant efficiency is adequate for the purpose. The energy comparison identified in this work is therefore a structural property of the configurations, not an artefact of the no-ERD assumption.

Integrating the device also produced two results that a post hoc estimate could not have obtained. Energy recovery attenuates the energy penalty of fouling, since without recovery the specific energy consumption of C2 rises by 1.02 kWh·m^−3^ over the horizon under severe fouling and with recovery by 0.53 kWh·m^−3^, roughly half of that cost being absorbed by the device because a substantial part of the additional pressure required is recovered from the concentrate rather than dissipated. Energy recovery and the product specification act in opposite directions: because recovery returns most of the pressurization energy, the penalty for operating at low recovery largely disappears and the energy optimum moves towards lower first-pass pressure and lower recovery, which yields a more concentrated first-pass permeate, so that under severe fouling the unconstrained energy optimum would deliver 64.1 mg·L^−1^ at the end of the horizon in C2, 61.8 in C1 and 34.7 in C3, against a specification of 20 mg·L^−1^. The system must therefore operate permanently away from its energy optimum in order to comply; the distance grows monotonically with the efficiency of the device, and improving energy recovery thus tightens the tension between energy efficiency and product quality. To our knowledge, this interaction has not been reported for two-pass trains supplying a polishing stage, and it qualifies the preceding observation: recovery absorbs the energy cost of fouling, but it does so by moving the system closer to its quality constraint.

### 4.4. Fouling-Driven Degradation: Comparison with Reported Field Data and Model Validation

The fouling scenarios adopted here are grounded in long-term field observations rather than arbitrary assumptions, thereby enabling meaningful comparison between modelled degradation and reported plant behaviour. The severe scenario combines a 25% decline in water permeability (αA = 0.25) with an 82% rise in solute permeability (αB = 0.82). The former matches the loss observed over extended operation of brackish-water FilmTec spiral-wound membranes by Abbas and Al-Bastaki [51]; the latter corresponds to the average increase in the solute-permeability coefficient B measured over 5026 days of full-scale BWRO operation by Chebil et al., who additionally reported increases of 88% and 87% in the ionic permeability of Cl^−^ and SO_4_^2−^, supporting the use of a single severity parameter for the three solutes [50].

The two independent field studies converge on this parameter: the same 500-day dataset that yields the water-permeability decline also reports an increase of about 85% in the solute-permeability coefficient [51], against the 82% measured over 5026 days by Chebil et al. [50], so that two plants of different feedwater, configuration and duration agree to within three percentage points on the magnitude of the rejection loss. A closer correspondence is available for seawater operation specifically, since a full-scale SWRO plant operated over 27 000 h across four years reported a decline of about 30% in the water-permeability coefficient together with an increase of about 70% in the solute-permeability coefficient [38]: the severe scenario of this work imposes 25% and 82% over 500 days, so that it reproduces the direction and approximate magnitude of measured seawater behaviour over a compressed horizon, with a somewhat milder permeability loss and a somewhat stronger rejection loss. The two field studies bracket the scenario rather than coinciding with it, which is the appropriate relationship for parameters intended to represent a plausible range rather than a specific installation.

The resulting energy penalty in C1—an SEC increase of up to 37.6% relative to the clean state under severe fouling—is broadly consistent with the 41–50% increase in SEC reported by Shouman et al. as the fouling factor falls from 1.0 to 0.6 [29], the modest difference being attributable to the different fouling metric and to the configuration-dependent response identified above. In the two configurations of the controlled comparison, the penalty is considerably smaller, 13.8% in C2 and 10.4% in C3, which is itself a result: the arrangement of the second-pass vessels determines not only the level of energy consumption but the extent to which fouling raises it.

This agreement between modelled and reported magnitudes constitutes the primary validation strategy of the present work. Because the model is not calibrated against a single plant dataset but is instead built from independently reported transport coefficients and fouling rates, its credibility rests on the consistency of its predictions with multiple field and literature sources rather than on a single experimental fit. Four elements support this indirect validation: the transport model itself is the well-established solution–diffusion framework [48], with membrane permeabilities fixed to manufacturer datasheet values and further corrected against the manufacturer’s projection software (WAVE) to within ~3%; the fouling severity parameters are anchored to long-term field data [38,50,51]; the resulting system-level energy penalties fall within the ranges independently reported for no-ERD SWRO [54] and for fouling-induced SEC increases [29]; and the numerical behaviour of the model is verified independently of its physical agreement, through the grid-convergence, solver-independence and physical-consistency checks reported in Section 2.7, so that the residual numerical uncertainty is quantified at 0.009 to 0.077 kWh·m^−3^ and can be compared against the effects the model is used to resolve. While this approach does not replace dedicated experimental validation—acknowledged as a limitation in Section 4.9—and while convergence with field magnitudes validates the model at the level of aggregate response rather than of the individual mechanisms that produce it, the convergence of independent benchmarks lends confidence that the dynamic model captures the operationally relevant consequences of progressive fouling.

### 4.5. Robustness of the Results to the Modelling Assumptions

Three assumptions of the model could plausibly govern the comparison between configurations: the functional form of the degradation law, the restriction of fouling to the first pass, and the treatment of permeate flow as an outcome of the optimization rather than a fixed production target. Each was tested by relaxing it, and the results are reported in the Supplementary Material. The comparison survives all three, but not in the same way in each case, and the differences are informative.

The linear form of Equations (6) and (7) was replaced by a power law and by the two-stage superposition of exponentials, all three calibrated to the same end-of-horizon state so that the comparison isolates the trajectory rather than the accumulated magnitude [36,37]. The end-of-horizon specific energy consumption is identical under the three laws, as are the ordering of the configurations and the magnitude of the gap between them, which is a consequence of the calibration rather than a coincidence, since the energy consumption depends on the state of the membranes and that state is common at day 500 by construction. The power-law and linear forms are moreover nearly indistinguishable at this degradation magnitude, the maximum difference in the water-permeability coefficient between them being of the order of 3% at mid-horizon; this limitation is recognized in the source of the power-law form itself, since fitting it to the 500-day dataset of Abbas and Al-Bastaki gives a decline coefficient of 0.042, within the range compiled in the literature, yet the authors report that the power law does not reproduce the data well over the initial period and propose rational and exponential forms instead [51].

The trajectory is a different matter. The two-stage law, which concentrates degradation in the early horizon and is the form with the strongest empirical support for long-term operation [36], brings forward the onset of the quality constraint by 130 to 210 days depending on the configuration and scenario, and multiplies the accumulated compliance effort by a factor of 1.8 to 2.6. The distinction that follows delimits the scope of this work: for comparing configurations, which is its object, the functional form of the degradation is immaterial, whereas for predicting when a given installation will reach its product-quality limit it is not, and the two-stage form would then be the appropriate choice. This also qualifies the practical implication of Section 4.2, since the onset days reported there are conditional on the linear law and should be read as a relative ordering between configurations rather than as absolute predictions for a plant.

Fouling was also imposed on the second-pass membranes as a fraction of the first-pass severity, from zero to the extreme case in which they degrade as fast as membranes fed with raw seawater. The scale has no independent physical meaning at intermediate values, since there is no measured degradation rate for permeate-fed second-pass membranes against which it could be calibrated, and the mechanisms that would operate there, compaction and polymer ageing, differ from those the first-pass parameters describe; only the two ends are interpretable. C2 remains below C3 at zero imposed severity, 8.38 against 11.46 kWh·m^−3^, and at half the first-pass severity, 10.83 against 12.26, and the ordering reverses only in the extreme case, 14.12 against 13.02, which is the condition that membrane autopsies of two-pass seawater trains contradict [52].

The reversal has an identifiable mechanism, and it confirms the prediction made in Section 4.1. The cost of quality compliance rises by a factor of thirteen in C2 over the range examined, from 0.46 to 6.20 kWh·m^−3^, but only by a factor of four and a half in C3, from 0.39 to 1.78, because imposed second-pass degradation acts selectively on the feature that confers the advantage: the series vessel operates on the most concentrated stream of the second pass, so the additional permeate it recovers is the first to be spoiled when that membrane degrades, whereas C3 has no equivalent exposure precisely because it has no equivalent advantage. The result identifies where the advantage of the hybrid arrangement resides and under what conditions it would be lost, and is reported as a finding of the sensitivity analysis rather than merely as a caveat.

The permeate flow reported throughout is an outcome of the optimization, and Section 3.2 shows that it drifts upward by 5.6–13.0% in C2 and 5.8–27.0% in C3 over the horizon. Industrial plants are frequently required to sustain a fixed output, so the analysis was repeated with each configuration constrained to hold its own nominal day-zero production within ±2%: both sustain it throughout the horizon in every scenario and neither reaches an operational limit, but the gap between them widens from 37% under free flow to 71% under fixed production, because holding production fixed raises the end-of-horizon specific energy consumption by 13.6% in C2 and by 41.6% in C3 relative to the free-flow case. The mechanism is again the compliance cost, which reaches 4.96 kWh·m^−3^ in C3 against 1.43 in C2, since, prevented from increasing its output, the pure-parallel arrangement cannot recover permeate from its own concentrate, having no series stage, and must instead buy quality margin from the first pass at a cost that grows steeply as fouling advances. The assumption of variable permeate flow was therefore conservative with respect to the conclusion drawn. One qualification applies to this last analysis: it is restricted to the two configurations of the controlled comparison, since the capacity of a C1 train exceeds theirs by a factor of two to three and a common plant-level comparison would require a different train-count basis. The plant-level consequences are taken up in Section 4.8.

### 4.6. Robustness of the Comparison to Parameter Uncertainty

Because the fouling severity parameters αA and αB are fixed a priori from long-term field data rather than fitted to a specific plant, it is essential to establish that the central findings do not depend on their exact values. The factorial sensitivity analysis in Section 3.6 addresses this concern directly by perturbing each parameter by ±25% around the severe-fouling base case—a range representative of the uncertainty involved in transferring fouling coefficients between membrane systems and operating conditions. Four conclusions follow.

First, the finding that defines the study is preserved across the entire perturbation range: C2 remains more energy-efficient than C3 in all five parameter combinations, so the efficiency difference between the two arrangements is not an artefact of the parameter values adopted but a structural property of the configurations. The magnitude of the difference is another matter, and is treated below.

Second, the two parameters act additively over the range examined, the interaction term being −0.012 kWh·m^−3^ in C2 and 0.029 in C3, that is, 1.0% and 4.9% of the corresponding main effects. This has a methodological consequence worth stating, since within this range the response can be described by main effects alone, so that perturbing each parameter separately, while unable to demonstrate the absence of interaction, does not in fact miss any; the factorial design is what establishes that, and it could not have been established otherwise.

Third, the analysis clarifies which physical mechanism governs each configuration’s dominant response, and the answer differs between them. In C2, the solute-permeability parameter dominates, with a main effect of 1.171 kWh·m^−3^ against 0.430 for the water-permeability parameter, whereas in C3 the ordering reverses, 0.404 against 0.599. This is mechanistically coherent with Section 4.2: C2 operates against the product specification, so degradation of salt rejection affects it directly, and its energy consumption follows αB, whereas C3 divides the feed among three branches, operates each vessel at lower recovery and retains more quality margin, so that what limits it is the loss of water permeability and its energy consumption follows αA. The two configurations are thus vulnerable to different degradation mechanisms, which has a practical consequence for membrane selection and monitoring, since a plant operating the hybrid arrangement should track salt passage as its leading indicator whereas one operating the pure-parallel arrangement should track normalized permeability; it also bears on mitigation, as Section 4.8 notes, since a cleaning strategy that restores water permeability at the cost of rejection would benefit the two unequally.

Fourth, what is robust is the ordering and not the magnitude. The gap between the configurations ranges from 27.8% to 42.5% across the perturbation box, against 36.9% at the centre point, and the end-of-horizon uncertainty bands are 19.1% of the central value in C2 and 8.7% in C3, so that reporting the gap as a single figure would overstate the precision available. The asymmetry between the two bands is itself informative and follows from the third conclusion, since C2 is the more sensitive of the two because it is governed by the parameter that carries the wider relative perturbation. Distinguishing the two sources of uncertainty is what makes these figures interpretable: the numerical uncertainty of the model, arising from the discretization of the operating grid and quantified in Section 2.7, is 0.04 to 0.08 kWh·m^−3^, whereas the parametric uncertainty is 1.00 to 1.60 kWh·m^−3^, larger by more than an order of magnitude. The dominant source of uncertainty in this model is therefore the value of the fouling parameters and not the numerics, which has a direct implication for where further effort would be worthwhile, since refining the numerical scheme would not improve the reliability of the predictions whereas measuring the degradation coefficients of a specific installation would. Both sources remain below the difference between the configurations, 3.09 kWh·m^−3^ at the end of the horizon under severe fouling, which is the condition under which a comparative modelling study can support a design conclusion at all.

### 4.7. Relation to Existing Models and the Contribution of This Work

Dynamic modelling of fouling in reverse osmosis is an active field, but the existing literature operates at scales and scopes that differ from those of the present study. A first group of works resolves the temporal evolution of foulant accumulation coupled to the local flow field, but does so at the scale of a single channel or membrane element, typically through computational fluid dynamics, without addressing system-level energy or configuration [66]. A second group develops hybrid mechanistic–data-driven models that track time-evolving permeability and solute passage, but for a single fixed plant layout, with the aim of prediction and calibration against operational data rather than architectural comparison [40]. A third group, including the work most closely related to ours, analyses how the placement of modules within a single pressure vessel modulates the impact of permeability loss on the minimum-SEC operating point, again within a fixed train [8]. More recently, superstructure-based multi-objective optimization has compared multiple RO configurations on energy, cost, and environmental criteria [42], yet under steady-state design conditions rather than progressive fouling.

A body of work more directly adjacent to the present one has established the analysis of operating windows: the identification of the operating points satisfying manufacturer constraints, the selection among them of the minimum-energy point, and the comparison of alternative vessel configurations on that basis [8,41]. The concept of the operating window is therefore not a contribution of this work, and we cite it as prior art. What the present analysis adds is its evolution under progressive degradation, and specifically the separation of the technically feasible window from the narrower window that also satisfies a product specification, which is what Section 4.2 reports and which, to our knowledge, has not previously been resolved.

A complementary body of work addresses the specific context of hydrogen production by coupling SWRO with PEM electrolysis. However, these studies treat desalination essentially as a black box characterized by a fixed specific energy (commonly 3.5 kWh·m^−3^) or by a steady-state exergy balance, focusing their detail on the electrolyzer or on system-level techno-economics [17,19]; review-level treatments likewise discuss the SWRO–PEM coupling conceptually without resolving the desalination process under degradation [67]. The two-step route of reverse osmosis followed by conventional electrolysis is now widely regarded as the pragmatic pathway for large-scale hydrogen production [68], making the detailed behaviour of the desalination stage increasingly relevant.

Against this background, the contribution of the present work is to bring together four elements that, to the authors’ knowledge, have not previously been combined, as summarized in Table 9: a comparison of complete second-pass configurations, two of which are controlled for membrane type, element count and installed area, rather than a single fixed layout; progressive, time-dependent fouling represented through coupled A(t) and B(t) coefficients; the operating point re-optimized at every instant subject to a product specification; and the separation of the technically feasible operating window from the window that also satisfies that specification, followed over time. Table 9 reports for each study the system and membranes modelled, the modelling approach, whether the transport coefficients evolve in time and under what law, the horizon, the performance indicators reported and the constraints imposed, so that the basis of the comparison is quantitative rather than binary. As Table 9 shows, each representative prior study addresses only part of this combination; none integrates all four.

Two of these elements deserve comment because they are less commonly reported than the others. The first is the control for membrane area, since comparisons of RO configurations frequently differ simultaneously in topology, membrane type, and installed area, which confounds the effect of the arrangement with that of the hardware, and holding the latter fixed is what allows the 36.9% to 41.6% difference reported here to be attributed to the arrangement alone. The second is the coupling of the energy optimization to a product-quality constraint: where the desalination stage supplies potable water, quality is generally satisfied with a wide margin and the optimization is effectively unconstrained in that dimension, whereas where it supplies a polishing stage feeding an electrolyzer, the specification is tight enough to bind, and Section 4.2 shows that it is the constraint that binds first. Two limitations of the comparison in Table 9 should also be noted: the studies compared differ in system, membranes, feed and objective, so the table documents the scope of each rather than ranking them; the hybrid approach of Gaublomme et al. represents fouling not only through the two transport coefficients but also through the feed-spacer channel height [40], and therefore captures a mechanism the present model does not, as acknowledged in Section 4.9.

### 4.8. Design Implications for Desalination–Electrolysis Facilities

The results reported here concern a single train. Because reverse-osmosis capacity is reached by replicating trains rather than by scaling one [20,21,22], the plant-level consequences follow by normalization, and it is at that level that the differences between configurations acquire their practical magnitude. Normalizing the fixed-production results of Section 4.5 to a reference plant delivering 100 m^3^·h^−1^, with each train operating at its own design point under severe fouling, the pure-parallel configuration requires 43% more trains and therefore 43% more installed membrane area, 62% more seawater intake and 71% more installed pumping power than the hybrid one, for the same delivered product. These are physical quantities obtained without economic assumptions, and they correspond to the three principal drivers of cost: installed area to capital expenditure of the membrane system, intake flow to the sizing of intake and pretreatment, and pumping power to operating cost, so that a reader may apply their own price assumptions to them. One conclusion is robust to any price set: the industrial reference requires 3.7 times the second-pass membrane area and approximately twice the seawater intake of the hybrid configuration, in addition to consuming more energy per unit of product, and no plausible combination of prices reverses that ordering. A full levelized-cost assessment, incorporating membrane replacement, maintenance, and site-specific energy prices, is identified as the natural continuation of this work in Section 5.

The choice between arrangements is not settled by energy efficiency alone, because the two operational criteria identified in Section 4.2 do not select the same one. Where continuity of production at design efficiency is paramount, which is the case when the desalination stage feeds an electrolysis plant whose own economics depend on high utilization, the relevant criterion is the onset of the compliance cost, and the hybrid arrangement sustains its unconstrained optimum about 1.8 times longer under severe fouling; where maintenance intervals are fixed by other considerations and the plant will operate away from its optimum for part of each cycle, the relevant criterion is instead the rate at which that cost accumulates, and the hybrid arrangement accumulates it about twice as fast. The two criteria are reconciled by scheduling rather than by architecture, since the hybrid arrangement is preferable in both cases provided cleaning is scheduled before the onset, and it is only when cleaning cannot be scheduled that the comparison becomes ambiguous.

Two further implications follow from the mechanisms identified. The first, from Section 4.1, is that the second-pass element must be sized against the permeate flow the first pass delivers at its own optimum, since an oversized head element does not merely underperform locally but forces the first pass to overproduce, and the penalty is paid in intake and pumping power from the first day of operation. The second, from Section 4.6, is that the two arrangements are governed by different degradation parameters, so a plant operating the hybrid arrangement should monitor salt passage as its leading indicator while one operating the pure-parallel arrangement should monitor normalized permeability. The same asymmetry bears on cleaning strategy, since chemical cleaning in place partially restores the water-permeability coefficient while progressively degrading rejection through oxidative damage to the polyamide layer over repeated cycles, so that a mitigation which trades rejection for permeability would benefit the two arrangements unequally, favouring the configuration governed by αA and penalizing the one governed by αB.

Finally, the desalination stage examined here does not supply the electrolyzer directly; it supplies the polishing unit that precedes it, and the specification it must meet is that unit’s inlet requirement. Two consequences follow for the design of an integrated facility. The relevant coupling between desalination and electrolysis runs through the polishing stage, so that a desalination train operating closer to its specification transfers a larger ionic load to the ion-exchange or electrodeionization unit, shortening regeneration intervals and increasing consumable demand; the energy saved upstream by operating nearer the limit is therefore not saved by the facility as a whole, and the trade-off is between the pumping energy of the second pass and the regeneration burden of the polishing stage, whose quantification requires a model of the polishing unit that lies outside the boundary adopted here and is identified as future work. And Section 4.3 shows that improving the recovery device moves the energy optimum towards lower first-pass recovery and hence towards a more concentrated permeate, so that the system operates further from its energy optimum in order to comply; in a facility where the desalination stage is a small fraction of the total energy demand, often below 0.15% of the electrolysis input [17], the case for maximizing recovery efficiency in isolation is correspondingly weak, and the design objective is better stated as delivering the specification reliably at acceptable cost than as minimizing the specific energy consumption of the desalination stage.

### 4.9. Limitations

Several limitations should be acknowledged, and they fall into three groups.

Regarding the physical representation, the model transports the three ions that govern osmotic pressure and product quality, sodium, chloride and sulfate, and does not include the calcium, barium, strontium, carbonate and silica species, nor the acid–base chemistry, required to evaluate mineral saturation indices, so that the argument of Section 2.3 for restricting fouling to the first pass is one of comparative dilution against a stream known to operate without scaling and not a saturation-index calculation. The fouling representation is a quasi-steady-state, lumped description through the severity parameters αA and αB applied to Pass 1; it does not resolve the spatial distribution of foulant within elements, nor distinguish among fouling mechanisms (scaling, organic, biological), each of which can affect A and B differently [40], and it does not capture the change in channel hydrodynamics that foulant accumulation produces, which hybrid approaches representing the feed-spacer channel height explicitly do capture [40]. Compaction and polymer ageing of the second-pass membranes are likewise not represented; these are not fouling mechanisms and are expected to be small given that the second pass operates at roughly one ninth of the first-pass feed pressure, but they are not zero. The model also represents degradation as a passive process, with no maintenance intervention over the horizon: the qualitative direction of chemical cleaning in place is discussed in Section 4.8, but quantifying it would require a cleaning-frequency and recovery-efficiency submodel. Data-driven approaches to flux prediction offer a complementary route where transport is difficult to parameterize mechanistically, as in effluents whose fouling behaviour resists first-principles description [70].

Regarding the numerical scheme and the decision rule, the optimization is sequential rather than joint, since the Pass-1 operating point is selected on its own energy and the Pass-2 pressure is then optimized for the selected point, so that the result is not necessarily the global minimizer of the total specific energy consumption; the comparison between configurations is unaffected, since all are evaluated under the identical rule, but the absolute values should be read with this in mind. The operating point is re-evaluated independently at each time step, so the model imposes no continuity of the operating trajectory between consecutive steps, whereas a real plant adjusts gradually. And the energy accounting is bounded at the membrane train, since intake, pretreatment, external piping, post-treatment, and the polishing stage lie outside it, so that the reported figures are not plant-level specific energy consumptions and should not be compared directly with values that include the balance of plant.

Regarding the scope of the study, the analysis considers three configurations, selected as industrially representative arrangements of the second pass rather than as a survey of the design space, so that the extension to a larger number of vessels and to other topologies remains open, and the fixed-production analysis is further restricted to the two arrangements controlled for membrane area. It considers three discrete fouling scenarios—extended by the ±25% factorial perturbations—rather than a continuous distribution of field conditions, and a single feed composition at a single temperature. The product specification is imposed as a fixed limit of 20 mg·L^−1^, whereas the inlet requirement of a polishing stage depends on the technology adopted, on its regeneration regime and on the design of the train as a whole, so that the value used here should be read as a representative specification rather than a universal one. No economic assessment is performed: a levelized cost of water would require capital costs, replacement schedules, maintenance regimes, plant lifetime, discount rate and site-specific energy prices, none of which is modelled, and adopting representative values for all of them would make the cost ranking a function of those assumptions rather than of the physics this work addresses, so that the physical drivers of cost are reported instead in Section 4.8. Finally, the model is validated indirectly by convergence with independently reported field magnitudes as set out in Section 4.4, and not against a dedicated experimental campaign on the specific configurations modelled.

## 5. Conclusions

This work introduced a dynamic model of a two-pass SWRO system supplying the polishing stage of an electrolytic hydrogen plant, and used it to compare three second-pass configurations under progressive membrane fouling, with the operating point re-optimized at each instant to minimize specific energy consumption subject to a product-quality constraint. Two of the configurations share membrane type, element count, and installed area and differ only in the arrangement of their pressure vessels; the third, built on a larger head element, is the conventional industrial reference.

The central finding is that the choice of train architecture is not energetically neutral once fouling is considered. With every other design variable held fixed, the pure-parallel arrangement consumes between 36.9% and 41.6% more energy per unit of product than the parallel-plus-series one across the three fouling scenarios, and the difference widens to 71% when production is held fixed rather than allowed to drift. The mechanism is identified: a vessel placed in series recovers additional permeate from the concentrate of the parallel stage at residual pressure and therefore at essentially zero marginal energy, which raises recovery without raising pump duty. The advantage is consequently structural rather than incidental, and the conditions under which it would be lost, namely degradation of the series vessel itself or a series stage requiring its own pressurization, are identified and tested.

The second finding concerns which constraint limits such a system as its membranes degrade. Following the feasible operating window over time, and separating the pressures admitted by the manufacturer’s technical limits from those that also satisfy the product specification, shows that the technical window widens as fouling advances while the quality window contracts and closes: progressive fouling constrains this system through separation and not through hydraulics, and two configurations exhibit the behaviour independently. The operational consequence is measured by the cost of quality compliance, the energy penalty of remaining within specification, which reports both when compliance begins to cost and how rapidly that cost accumulates thereafter; the two are not ordered alike between configurations, and reporting only one would misrepresent the comparison.

A ±25% sensitivity analysis confirmed that the ordering of the configurations is preserved in every parameter combination, while placing the magnitude of the difference between 27.8% and 42.5%, and showed that the two arrangements are governed by different degradation parameters—rejection loss in the hybrid arrangement, permeability loss in the pure-parallel one—which has direct consequences for monitoring and cleaning strategy. Three alternative degradation laws leave the comparison unchanged while shifting the onset of the compliance cost by up to 210 days, so the architectural conclusion is insensitive to the functional form while the timing is not. Integrating an energy-recovery device reduces the specific energy consumption by 63–64% and preserves the ordering throughout the commercial range of device efficiencies, so that its significance lies not in the absolute energy figures but in the demonstration that progressive fouling discriminates between configurations that appear equivalent under clean, steady-state conditions.

For integrated desalination–electrolysis hubs, the practical implication is that three design consequences follow. The second-pass element should be sized against the permeate flow the first pass delivers at its own optimum, since an oversized head element forces the first pass to overproduce and the penalty is paid upstream from the first day of operation. Cleaning should be scheduled against the onset of the compliance cost rather than in response to a rising energy bill, since the model anticipates that instant at the design stage and bounds its uncertainty. And because the desalination stage supplies the polishing unit rather than the electrolyzer, operating nearer the specification transfers ionic load downstream, so the design objective is better stated as delivering the specification reliably at acceptable cost than as minimizing the energy of the desalination stage in isolation. Future work will incorporate an explicit model of the polishing stage, spatially resolved and mechanism-specific fouling, active cleaning cycles, and a levelized-cost assessment under site-specific conditions.

## Figures and Tables

**Table 1:** Seawater feed composition and operating conditions. The product-quality constraint applies to the final permeate of the second pass and corresponds to the maximum total dissolved solids (TDS) admitted at the inlet of the downstream polishing stage, not to the electrolyzer specification; the treatment train and its successive quality requirements are described in the Introduction.

Parameter	Symbol	Value	Unit	Description
Sodium concentration in feed	C_Na,f_	12.935	kg·m^−3^	Seawater composition
Chloride concentration in feed	C_Cl,f_	17.893	kg·m^−3^	Seawater composition
Sulfate concentration in feed	C_SO4,f_	2.781	kg·m^−3^	Seawater composition
Feed water density	ρ	1017	kg·m^−3^	At 25 °C
Operating temperature	T	25	°C	Isothermal
Temperature correction factor	TCF	1.000	—	Unity at the reference temperature
Membrane ageing factor	F_f_	0.85	—	Manufacturer guideline
Pump efficiency	ηp	0.85	—	Both passes
Maximum TDS at polishing inlet	Cp,max	0.020	kg·m^−3^	Constraint on final permeate
Simulation horizon	T_h_	500	d	51 time points
Time step	Δt	10	d	—
First-pass pressure grid	—	30 to 70, step 0.2	atm	Refined; see Section 2.7
First-pass feed-flow grid	—	3.0 to 14.1, step 0.10	m^3^·h^−1^	Refined; see Section 2.7
Second-pass pressure grid	—	3 to 10, step 0.02	atm	—
Energy-recovery efficiency	ηERD	0.96	—	Isobaric device; 0 in the base case

**Table 4:** Verification of the numerical core of the model. Each item states what was tested, how, and the outcome. The residual numerical uncertainty reported in the last row is the quantity against which the parametric uncertainty reported in Section 3.6 should be compared.

Item	Test	Outcome
Grid convergence	Refinement of the first-pass operating grid by a factor of 7.5 in the number of nodes	End-of-horizon SEC changes by 0.5%; amplitude of the non-monotonic excursions falls by a factor of 5.4, from 0.19 to 0.04 kWh·m^−3^
Solver independence from the initial estimate	Element-level system re-solved from an initial estimate adapted to the low-salinity second-pass feed	Same root to within a relative difference of 7 × 10^−8^
Physical consistency	Computed permeate flow against applied pressure over the admissible range	Linear, with successive increments of 0.119, 0.119 and 0.118 m^3^·h^−1^ per 2 atm
Concentration-polarization bound	Upper bound on the argument of the polarization expression, Equation (3)	Would require an element recovery of 429% against manufacturer limits of 10% and 30%; the bound is inactive
Permeability correction	Empirical correction evaluated over the full operating domain	Both factors positive and bounded; product between 0.773 and 1.225
Polarization factor	Range attained over the nine simulations	1.051 to 1.062, against a maximum admissible value of 1.073; correction of 5 to 6% on the osmotic pressure
Residual numerical uncertainty	Largest non-monotonic excursion of the SEC time series after refinement	0.009 to 0.077 kWh·m^−3^ across the nine simulations

**Table 5:** Comparative performance of configurations C1, C2, and C3 across fouling scenarios at the beginning and at the end of the 500-day horizon. Specific energy consumption refers to the complete train and is expressed per unit of final permeate. Recovery refers to the total first-pass seawater feed. Flux is the mean permeate flux of the second pass. The cost of quality compliance is the difference between the minimum total specific energy consumption with and without the product-quality constraint imposed, evaluated over the same set of candidate operating points, and is therefore non-negative by construction; the last column gives the day on which it first becomes non-zero.

Config.	Scenario	SEC Day 0	SEC Day 500	Increase (%)	Product Flow	Recovery (%)	Flux	Seawater Feed	Quality Cost	Onset Day
C1	Low	7.57	8.03	6.1	3.72	23.3	20.7	16.0	0.60	0
	Moderate	7.57	9.01	19.0	4.22	24.0	23.5	17.6	1.43	0
	Severe	7.57	10.42	37.6	4.59	22.7	25.5	20.2	2.92	0
C2	Low	7.36	7.50	2.0	1.96	20.0	40.4	9.8	0	—
	Moderate	7.36	7.74	5.3	2.07	20.7	42.7	10.0	0	—
	Severe	7.36	8.38	13.8	2.09	20.1	43.2	10.4	0.46	310
C3	Low	10.38	10.56	1.7	1.37	14.0	28.3	9.8	0	—
	Moderate	10.38	10.97	5.6	1.49	14.6	30.7	10.2	0.08	320
	Severe	10.38	11.46	10.4	1.65	15.0	34.0	11.0	0.39	170

Note: SEC and quality cost in kWh·m^−3^; product flow and seawater feed in m^3^·h^−1^; flux in L·m^−2^·h^−1^; onset day in d. C2 and C3 share membrane type, element count, and installed area (48.48 m^2^) and differ only in the arrangement of the vessels; C1 installs 179.76 m^2^ and is reported as the industrial reference. An em dash in the last column indicates that the quality constraint never becomes binding within the horizon. The onset is defined as the first time point at which the cost of quality compliance remains non-zero over three consecutive steps; isolated single-step values below the numerical resolution of the model are not treated as an onset.

**Table 6:** Effect of energy recovery on the specific energy consumption at the end of the horizon under severe fouling. A pressure-exchanger-type device is integrated into the operating-point selection rather than applied as a post-calculation: the specific energy consumption of every candidate first-pass operating point is recomputed with recovery before the candidates are ranked. Recovery is applied to the first pass only, since the second operates between 3 and 10 atm and its concentrate carries no significant recoverable energy.

Configuration	Without Recovery (kWh·m^−3^)	With Recovery (kWh·m^−3^)	Reduction (%)	Product Flow (m^3^·h^−1^)	Recovery (%)
C1	10.415	3.796	63.6	4.49	19.0
C2	8.376	2.999	64.2	2.10	15.0
C3	11.464	4.217	63.2	1.60	11.8

**Table 7:** Sensitivity to the efficiency of the recovery device, at the end of the horizon under severe fouling, across the range spanned by commercial technologies: reported efficiencies range from 75–85% for centrifugal devices to 95–97% for isobaric chambers [56]. The two configurations compared at equal membrane area are evaluated at four efficiencies.

η of the Device	Technology	C2 (kWh·m^−3^)	C3 (kWh·m^−3^)	Gap (%)	Reduction (%)
0	No recovery	8.376	11.464	36.9	—
0.85	Centrifugal, maximum	3.748	5.106	36.3	55.3
0.90	Pelton turbine, turbocharger	3.446	4.723	37.1	58.9
0.96	Isobaric chamber	2.999	4.217	40.6	64.2

Note: The gap between the two configurations varies by 4.3 percentage points across the entire commercial range of device efficiencies, compared with 27.8 to 42.5% for the parametric uncertainty of the fouling coefficients reported in Section 3.6. The em dash in the Reduction column denotes the no-recovery case, which is the baseline against which the reductions are computed and therefore has no reduction value.

**Table 8:** Joint sensitivity of both configurations to the two fouling severity parameters, evaluated as a two-level full factorial with a centre point on a ±25% perturbation box around the severe-fouling base case (αA = 0.25, αB = 0.82). The upper block gives the end-of-horizon specific energy consumption at each combination; the lower block gives the main effects and the interaction term computed from the four corners.

Case	αA	αB	C2 (kWh·m^−3^)	C3 (kWh·m^−3^)	Gap (%)
Centre point	0.2500	0.8200	8.376	11.464	36.9
αA− αB−	0.1875	0.6150	7.735	11.022	42.5
αA+ αB−	0.3125	0.6150	8.177	11.592	41.8
αA− αB+	0.1875	1.0250	8.918	11.397	27.8
αA+ αB+	0.3125	1.0250	9.336	12.025	28.8
Band width	—	—	1.601 (19.1%)	1.003 (8.7%)	27.8 to 42.5
Main effect of αA	—	—	0.430	0.599	—
Main effect of αB	—	—	1.171	0.404	—
Interaction	—	—	−0.012 (1.0%)	0.029 (4.9%)	—

Note: The interaction term is 1.0% and 4.9% of the corresponding main effects, so the two parameters act additively over the range examined. The two configurations are nevertheless dominated by different parameters, and the ordering between them is preserved in all five combinations. Em dashes indicate cells that do not apply: the parameter columns for the summary rows (band width, main effects, interaction) and the gap column for the derived-effect rows.

**Table 9:** Comparison of the present work with representative recent RO fouling and optimization modelling studies. For each study, the table reports the system and membranes modelled, the modelling approach, whether the transport coefficients evolve in time and under what law, the time horizon, the performance indicators reported, and the constraints imposed.

Study	System and Membranes	Approach	Time-Dependent Coefficients	Horizon	Indicators Reported	Constraints Imposed
Ruiz-García et al. [8]	BWRO, module position within a single vessel	Steady-state simulation	No	—	SEC, optimal operating point	Manufacturer limits
Ruiz-García & Carrascosa-Chisvert [41]	BWRO, two vessel configurations (2:1 and 3:2)	Steady-state simulation	No	—	SEC, recovery, operation windows, optimal operating points	Manufacturer limits
Ruiz-García & Nuez [36]	BWRO full-scale plant	Fitting of permeability decline	Yes, superposition of two exponentials	10 years	Decline of A	—
Gaublomme et al. [40]	Full-scale RO, hybrid mechanistic and data-driven	Data-driven membrane resistance coupled to solution–diffusion	Yes, through A, B, and feed spacer channel height	Calibrated on plant data	Membrane resistance, permeability, pressure drop	Not reported
Ling et al. [66]	Single membrane channel	CFD; transient Navier–Stokes with adsorption–desorption	Yes, through foulant accumulation	0.5 h	Permeate flux, pressure drop, fouling pattern	—
Lee et al. [17]	Water and energy technologies, RO coupled to electrolysis	Process and economic analysis	No	—	Levelized cost of water; technology comparison	—
León Zerpa & Ramos Martín [38]	SWRO full-scale plant	Model fitting to plant data	Yes, three models compared	27 000 h	A decline of 30%, B increase of 70%	—
Lim, Ki & Lim [69]	SWRO, single pass, design configurations	Simulation under time-variant feed	No	Not reported	SEC under constant-permeate operation	Not reported
Nour-Mohammad & Fakhroleslam [42]	SWRO superstructure with pressure exchanger	Multi-objective optimization	No	—	SEC, specific water cost, recovery	Not reported
Fang [62]	SWRO with energy recovery	Genetic-algorithm optimization	No	—	SEC, optimal recovery	Not reported
This work	Two-pass SWRO, three second-pass configurations	Dynamic quasi-steady simulation with re-optimization at every step	Yes, three laws compared	500 d, 51 points	SEC, recovery, flux, seawater feed, cost of quality compliance, operating windows	Manufacturer limits and product-quality specification

Note: Operating-window analysis of reverse-osmosis systems, including the comparison of alternative vessel configurations and the identification of optimal operating points by minimum specific energy consumption, has been established by Ruiz-García and co-workers. The contribution of the present work is not the concept of the operating window but its evolution under progressive membrane degradation, and, in particular, the separation of the technically feasible window from the window that also satisfies the product-quality specification. Throughout the table, an em dash indicates that the field does not apply to the study (for example, a steady-state analysis has no temporal horizon), whereas ‘Not reported’ indicates a field that could apply but is not specified in the source.

## Data Availability

The original contributions presented in this study are included in the article/Supplementary Material. The MATLAB model code developed for this work is not readily available because it was developed in collaboration with an industrial partner. Requests to access the code should be directed to the corresponding author.

## Supplementary Materials

The following supporting information can be downloaded at https://www.mdpi.com/article/10.3390/membranes16090290/s1.

## Abbreviations

**Symbols**		
Aw	water permeability coefficient of the membrane	m·s^−1^·Pa^−1^
A0	initial (unfouled) water permeability coefficient	m·s^−1^·Pa^−1^
A(t)	time-dependent water permeability coefficient	m·s^−1^·Pa^−1^
A_m_	active membrane area per element	m^2^
Bi	solute permeability coefficient of species i	m·s^−1^
B(t)	time-dependent solute permeability coefficient	m·s^−1^
Cf,i	feed concentration of species i	kg·m^−3^
Cp,i	permeate concentration of species i	kg·m^−3^
Cr,i	reject concentration of species i	kg·m^−3^
Cp	permeate solute concentration	mg·L^−1^
Cp,max	maximum admissible TDS at the polishing inlet	kg·m^−3^
F_f_	membrane ageing factor	—
Jw	water flux	m·s^−1^
Js,i	solute flux of species i	kg·m^−2^·s^−1^
kfac	unit-conversion factor of the pump power expression	kW·(atm·m^3^·h^−1^)^−1^
Pf	feed (applied) pressure	atm
ΔP	transmembrane pressure	atm
Δπ	osmotic-pressure difference	atm
Qf	feed volumetric flow rate	m^3^·h^−1^
Qp	permeate volumetric flow rate	m^3^·h^−1^
Qr	reject volumetric flow rate	m^3^·h^−1^
T	operating temperature	°C
TCF	temperature correction factor	—
t	operating time	d
Y	element recovery	—
αA	severity parameter for water-permeability decline	—
αB	severity parameter for solute-permeability increase	—
β	concentration-polarization factor	—
ηp	pump efficiency	—
ηERD	efficiency of the energy-recovery device	—
π	osmotic pressure	atm
ρ	feed water density	kg·m^−3^
**Abbreviations**		
ASTM	American Society for Testing and Materials	
AWE	alkaline water electrolyzer	
B1, B2	high-pressure pump of Pass 1 and Pass 2, respectively	
BWRO	brackish water reverse osmosis	
C1	series Pass-2 configuration with an 8-inch head element (V2A → V2B)	
C2	parallel-plus-series Pass-2 configuration (V2A, V2B → V2C)	
C3	pure-parallel Pass-2 configuration (V2A, V2B, V2C)	
CFD	computational fluid dynamics	
EDI	electrodeionization	
ERD	energy recovery device	
ISO	International Organization for Standardization	
IX	ion exchange	
MF	microfiltration	
PEM	proton exchange membrane	
RO	reverse osmosis	
SEC	specific energy consumption (kWh·m^−3^)	
SWRO	seawater reverse osmosis	
TDS	total dissolved solids	
UF	ultrafiltration	
V2A, V2B, V2C	Pass-2 pressure vessels	
WAVE	Water Application Value Engine (manufacturer projection software)	
