# Supplementary material for: Second-Pass Vessel Arrangement and Separation-Limited Operation in Two-Pass Seawater Reverse Osmosis Under Progressive Fouling: Implications for Electrolytic Hydrogen Production
Source: Membranes (Basel). 2026 Aug 28;16(9):290. doi: 10.3390/membranes16090290 (PMC13608914; doi:10.3390/membranes16090290)
Supplement: Supplementary file 1 [file membranes-16-00290-s001.zip › Supplementary_Materials.pdf]

Supplementary Materials

Supplementary Figures and Figure Captions

Manuscript ID: membranes-4461946

The five figures below accompany the supplementary analyses. The first four verify that a conclusion of the manuscript does not depend on a specific modelling assumption; the fifth reports the time evolution of the specific energy consumption with and without energy recovery.

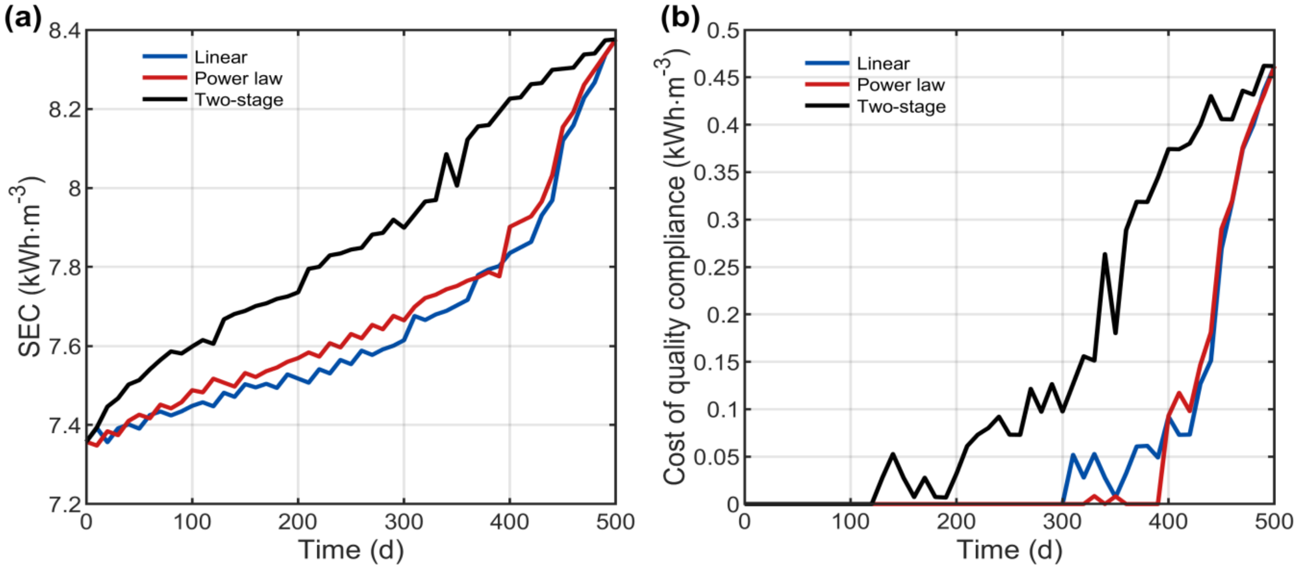

Figure S1. Results under the three functional forms implemented for the long-term decline of the water-permeability coefficient — linear, power law and two-stage superposition of exponentials — all calibrated to the same end-of-horizon state so that the comparison isolates the trajectory of the degradation rather than its accumulated magnitude. Configuration C2 under severe fouling. (a) Specific energy consumption over the horizon; the three curves converge at day 500 by construction. (b) Cost of quality compliance; the two-stage law brings forward the onset of the constraint and multiplies the accumulated compliance effort. Tabulated values are given in Table S1.

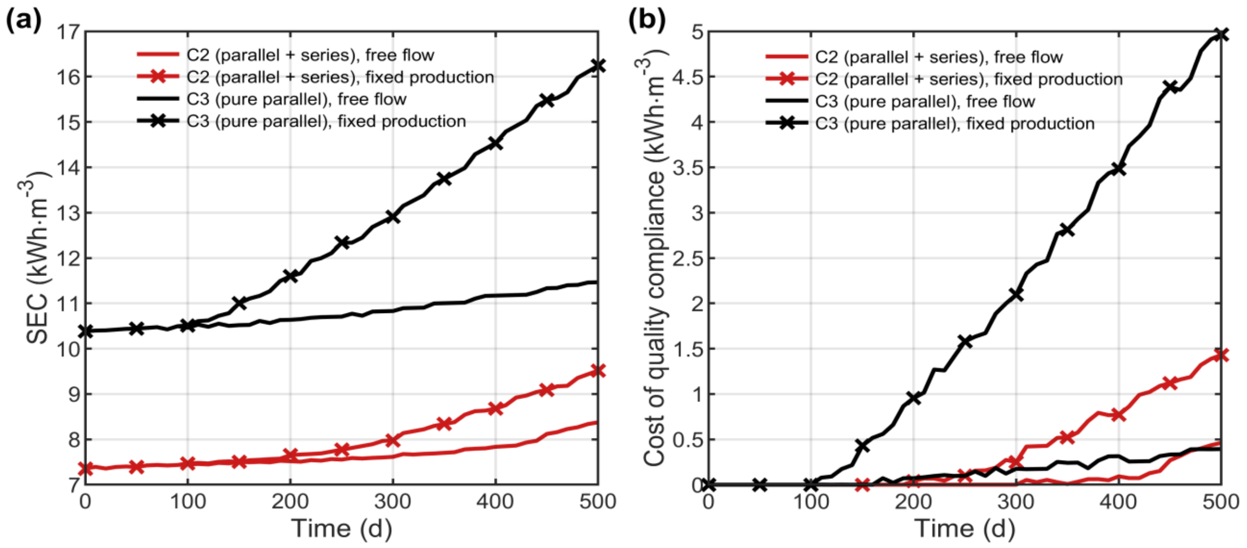

Figure S2. Operation at fixed production compared with free flow, for configurations C2 and C3 under severe fouling. Each configuration is constrained to sustain its own nominal day-zero output within a tolerance band of ±2%. (a) Specific energy consumption; (b) cost of quality compliance. Both configurations sustain their nominal production throughout the horizon; the gap between them widens from 37% under free flow to 71% under fixed production. Tabulated values are given in Table S2.

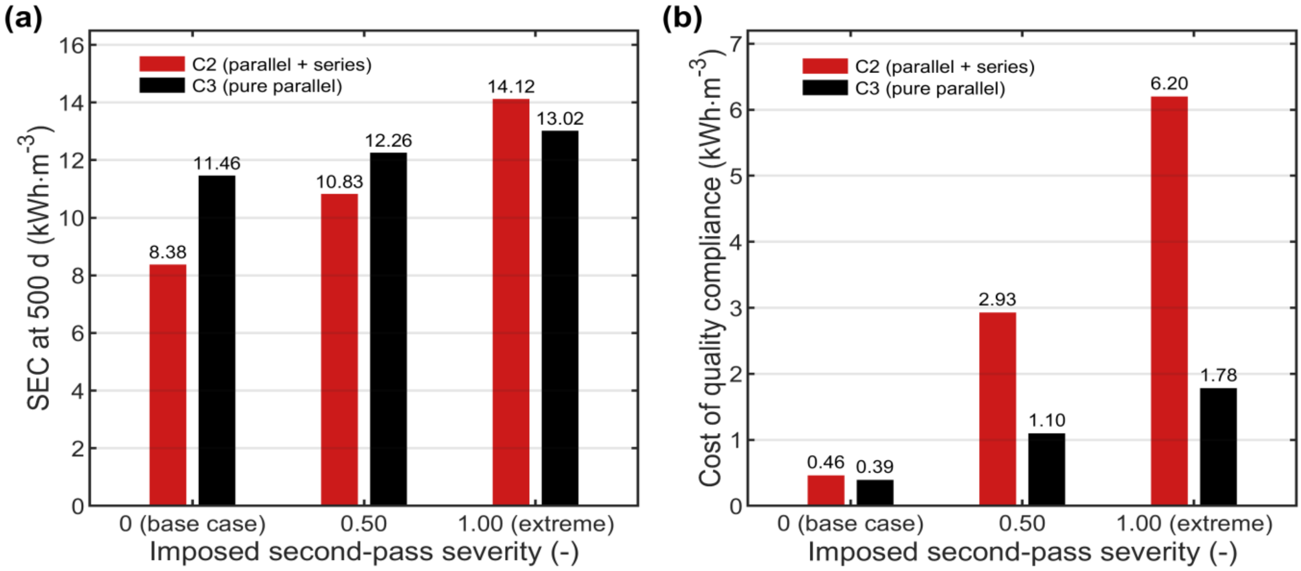

Figure S3. Sensitivity of the results to degradation imposed on the second-pass membranes, expressed as a fraction of the first-pass severity, for configurations C2 and C3 under severe first-pass fouling. (a) End-of-horizon specific energy consumption; (b) cost of quality compliance at the end of the horizon. The scale has no independent physical meaning at intermediate values: only its ends do. Zero is the model as reported and unity is the condition in which a membrane fed with permeate is assumed to degrade as fast as one fed with seawater, which published membrane autopsies of two-pass seawater trains contradict. Tabulated values are given in Table S3.

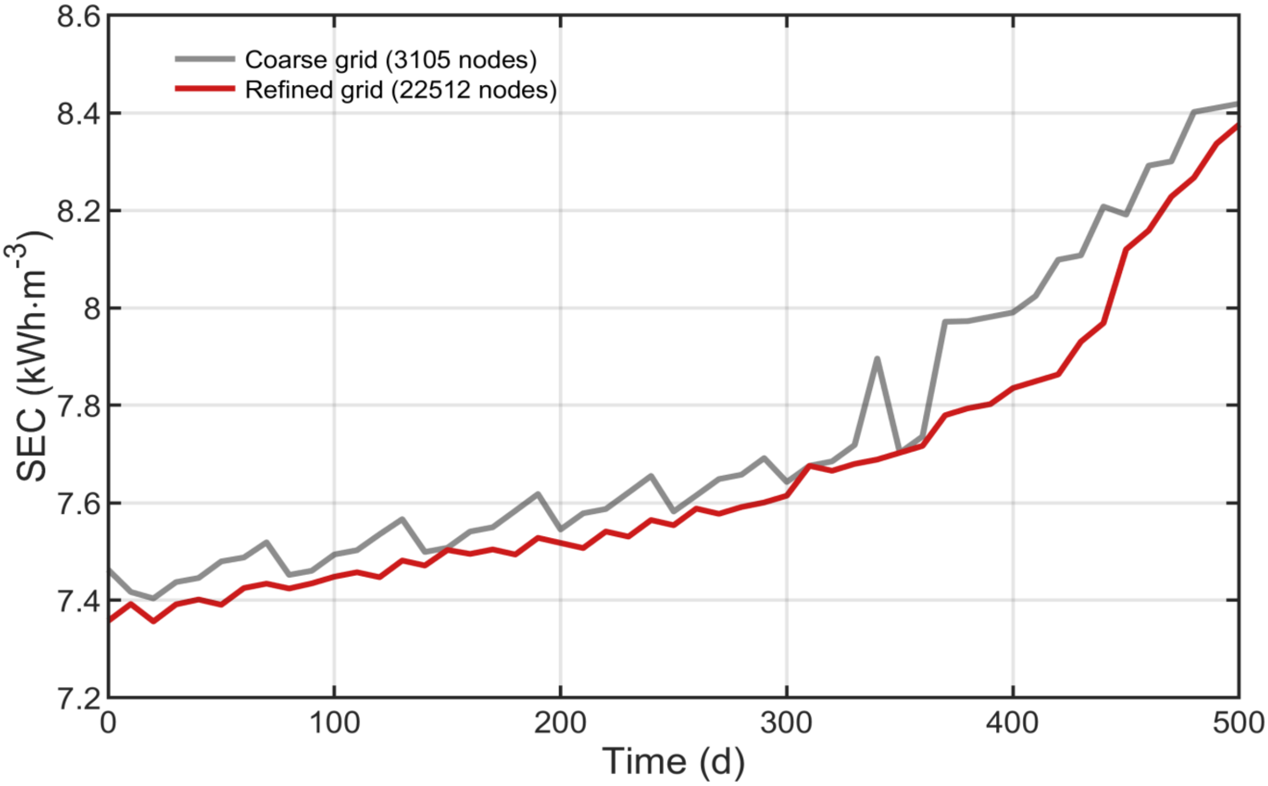

Figure S4. Grid-convergence study of the first-pass operating grid, for configuration C2 under severe fouling. Refining the grid by a factor of 7.5 in the number of nodes changes the end-of-horizon specific energy consumption by 0.5% and reduces the amplitude of the non-monotonic excursions by a factor of 5.4, from 0.19 to 0.04 kWh·m^{-3}. The proportionality between refinement and residual identifies the excursions as a discretization artefact. The refined grid is used throughout the manuscript.

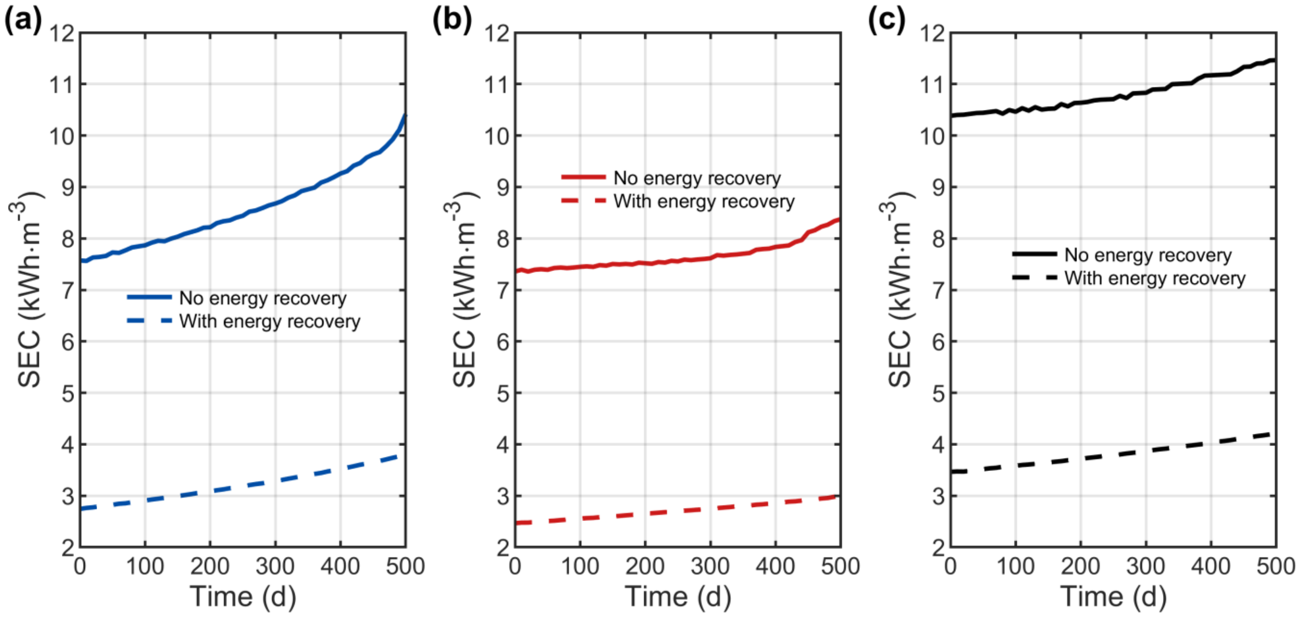

Figure S5. Specific energy consumption of the complete train over the 500-day horizon under severe fouling, with and without energy recovery: (a) configuration C1, series with an 8-inch head element; (b) configuration C2, two vessels in parallel discharging to a third in series; (c) configuration C3, three vessels in pure parallel. Solid lines give the base case without an energy-recovery device; dashed lines the case with a pressure-exchanger-type device at an efficiency of 0.96, integrated into the operating-point selection rather than applied as a post-calculation. The three panels share a common vertical scale. The reduction at the end of the horizon is 63.6%, 64.2% and 63.2% respectively, and the ordering between configurations is preserved with and without the device. End-of-horizon values are given in Table 6 of the main text.

Supplementary Tables

Manuscript ID: membranes-4461946

The analyses below verify that the conclusions do not depend on specific modelling assumptions. They are reported as supplementary material because none of them supports a claim of the paper: each tests whether a claim would survive a different assumption, and in each case it does. The plant-level normalization derived from Table S2 is reported in the Discussion, since it is a result rather than a verification.

Table S1. Results under the three functional forms reported in the literature for the long-term decline of the water-permeability coefficient. The three laws are calibrated to the same end-of-horizon state, so that the comparison isolates the trajectory of the degradation rather than its accumulated magnitude.

Quantity	Config.	Scenario	Linear	Power law	Two-stage
End-of-horizon SEC (kWh·m^{-3})	C2	Severe	8.376	8.376	8.376
	C3	Severe	11.464	11.464	11.464
Onset of quality cost (d)	C2	Severe	310	400	130
	C3	Moderate	320	310	110
	C3	Severe	170	180	40
Accumulated quality cost (kWh·m^{-3}·d)	C2	Severe	29.2	27.1	75.6
	C3	Moderate	14.4	15.3	30.2
	C3	Severe	68.9	61.5	122.1

The end-of-horizon specific energy consumption is identical under the three laws, as is the ordering of the configurations and the magnitude of the gap between them, so the comparative conclusion is insensitive to the functional form. The trajectory is not: the two-stage law, which concentrates degradation in the early horizon, brings forward the onset of the quality constraint by 130 to 210 days depending on the configuration and scenario, and multiplies the accumulated compliance effort by a factor of 1.8 to 2.6. The power-law and linear forms are nearly indistinguishable at this degradation magnitude.

Table S2. Operation under fixed production, with each configuration constrained to sustain its own nominal day-zero output within a tolerance band of ±2%, compared with the free-flow results of Table 5. Both configurations sustain their nominal production throughout the horizon in every scenario.

Config.	Scenario	Target (m^3·h^{-1})	SEC free (kWh·m^{-3})	SEC fixed (kWh·m^{-3})	Penalty (%)	Quality cost (kWh·m^{-3})	Seawater feed (m^3·h^{-1})
C2	Low	1.853	7.504	7.594	1.2	0	9.60
	Moderate	1.853	7.745	8.153	5.3	0.253	9.80
	Severe	1.853	8.376	9.517	13.6	1.430	10.60
C3	Low	1.297	10.563	10.770	2.0	0.078	9.80
	Moderate	1.297	10.967	13.239	20.7	2.218	10.80
	Severe	1.297	11.464	16.238	41.6	4.963	12.00

The permeate flow in the free-flow simulations does not oscillate; it drifts slowly and monotonically, rising by 5.6 to 13.0% in the hybrid configuration and by 5.8 to 27.0% in the pure-parallel one, because the operating point that minimizes specific energy consumption shifts towards higher pressure and higher output as the membranes degrade. Imposing fixed production does not weaken the comparison between configurations but strengthens it: under severe fouling the pure-parallel arrangement consumes 36.9% more energy per unit of product than the hybrid one with free flow, and 71% more under fixed production. Normalized to a reference plant of 100 m^3·h^{-1} with each train operating at its own design point, the pure-parallel configuration requires 43% more trains and installed membrane area, 62% more seawater intake and 71% more installed pumping power.

Table S3. Sensitivity of the results to degradation imposed on the second-pass membranes, expressed as a fraction of the first-pass severity. The scale has no independent physical meaning: only its ends do. Zero is the model as reported, and unity is the condition in which a membrane fed with permeate is assumed to degrade as fast as one fed with seawater, which published membrane autopsies of two-pass seawater trains contradict.

Second-pass severity (fraction of first pass)	C2 SEC ($kWh{\cdot}m^{-3}$)	C3 SEC ($kWh{\cdot}m^{-3}$)	Ordering	C2 quality cost ($kWh{\cdot}m^{-3}$)	C3 quality cost ($kWh{\cdot}m^{-3}$)
0 (as reported)	8.376	11.464	C2 < C3	0.462	0.393
0.50	10.827	12.258	C2 < C3	2.929	1.098
1.00 (extreme)	14.123	13.019	C3 < C2	6.200	1.783

The ordering is preserved over the physically supported range and reverses only in the extreme case. The cost of meeting the product specification rises by a factor of thirteen in the hybrid configuration and by a factor of four and a half in the pure-parallel one, which identifies the mechanism: the series stage of the hybrid arrangement operates on the most concentrated stream of the second pass, so the additional permeate it recovers is the first to be spoiled when that membrane degrades. The pure-parallel arrangement has no equivalent exposure because it has no equivalent advantage.

Table S4. Number of feasible second-pass feed pressures out of the 351 swept values, counted twice: subject only to the manufacturer's technical limits, and subject additionally to the product-quality specification. Severe fouling. The window is evaluated at the operating point that minimizes the first-pass specific energy consumption. This choice is deliberate: evaluated at the point the model actually selects there would be, by construction, at least one feasible pressure at every instant and the window could never close, so the quantity would be uninformative. Evaluated at the energy-optimal point it shows how many pressures would be available if the system could operate there, and therefore when it ceases to be able to.

Day (d)	C1 technical	C1 quality	C2 technical	C2 quality	C3 technical	C3 quality
0	0	0	147	122	56	41
50	0	0	150	107	58	25
100	0	0	160	108	65	24
150	0	0	162	90	66	6
200	0	0	167	75	71	0
250	0	0	171	55	74	0
300	0	0	174	24	77	0
350	0	0	175	0	77	0
400	0	0	189	0	87	0
450	0	0	190	0	88	0
500	0	0	194	0	92	0

The technically feasible window does not contract as the membranes foul — it widens, from 147 to 194 pressures in the hybrid configuration and from 56 to 92 in the pure-parallel one — whereas the window that also satisfies the quality specification contracts monotonically and closes, between days 300 and 350 in the hybrid configuration and between days 150 and 200 in the pure-parallel one. Progressive fouling therefore constrains this system through separation and not through hydraulics, and two configurations exhibit the behaviour independently. The conventional configuration has no feasible pressure at this operating point at any time: its head vessel exceeds the element recovery limit from the first day, so it must operate away from the first-pass energy optimum throughout the horizon, which is a different limitation and is discussed in Section 4.1.

Table S5. Best product quality attainable at the operating point that minimizes the first-pass specific energy consumption, under severe fouling, against the specification of 20 mg·L^{-1}. The value is the minimum over the swept second-pass feed pressures, with the quality constraint lifted.

**Day (d)**	**C2 (mg·L^{-1})**	**C3 (mg·L^{-1})**
0	15.12	17.23
50	16.17	18.44
100	16.31	18.55
150	17.34	19.71
200	18.11	20.59
250	18.90	21.49
300	19.72	22.38
350	20.85	23.65
400	20.21	22.95
450	21.13	24.01
500	21.58	24.50

The attainable quality crosses the 20 mg·L^{-1} specification between days 150 and 200 in the pure-parallel configuration and between days 300 and 350 in the hybrid one, which is when the corresponding quality window of Table S4 closes. From that point onwards the system must abandon the first-pass energy optimum in order to meet the specification, and the cost of doing so is the quantity reported in Table 5. Values for the conventional configuration are not given because its second pass admits no feasible pressure at this operating point, as shown in Table S4. The non-monotonic step between days 350 and 400 is a residual artefact of the discretization of the first-pass operating grid, quantified in Table 4.
